# Interactions of Antibody Drug Conjugate Anti‐Tubulin and Topoisomerase I Inhibitor Payloads with Radiotherapy to Potentiate Immunotherapy

**DOI:** 10.1002/advs.202506552

**Published:** 2025-10-24

**Authors:** Jacqueline Lesperance, Bryan S. Yung, Michael M. Allevato, Marcus M Cheng, Maria F. Camargo, Robert Saddawi‐Konefka, Kanika Dhawan, Ashwyn K. Sharma, Mahsa Mortaja, Sophie Bice, Daniel J. Scanderbeg, Diego Alvarado, Jyoti Mayadev, Ramez N. Eskander, Stephen R. Adams, Pippa F. Cosper, J. Silvio Gutkind, Sunil J. Advani

**Affiliations:** ^1^ Department of Radiation Medicine and Applied Sciences University of California San Diego La Jolla CA 92093 USA; ^2^ Department of Pharmacology University of California San Diego La Jolla CA 92093 USA; ^3^ Department of Otolaryngology‐Head and Neck Surgery University of California San Diego La Jolla CA 92093 USA; ^4^ Moores Cancer Center La Jolla CA 92093 USA; ^5^ Department of Surgery University of California San Diego La Jolla CA 92093 USA; ^6^ Department of Human Oncology University of Wisconsin Madison WI 53705 USA; ^7^ Celldex Therapeutics Hampton NJ 08827 USA; ^8^ Division of Gynecologic Oncology Department of Obstetrics Gynecology and Reproductive Sciences University of California San Diego La Jolla CA 92093 USA

**Keywords:** antibody drug conjugates, immunotherapy, radiosensitization, radiotherapy

## Abstract

The most effective treatments for locally advanced cancers rely on non‐targeted chemotherapies given with radiotherapy. Advances in cancer biology have identified vulnerabilities amenable to precision oncology approaches including antibody drug conjugates (ADCs). In theory, ADCs combine specificity of cancer cell receptor antibody targeting with potent cytotoxins. However, toxicities and resistance limit ADC clinical efficacy. Delivering ADCs with radiotherapy can improve their therapeutic index. Here, the combination of ADC payloads (anti‐tubulin monomethyl auristatin E (MMAE) or topoisomerase I inhibitors DXd and SN‐38) with radiotherapy is reported in immune‐competent murine models. To directly compare ADC payload effects and remove targeting bias, the payloads are tested as free drugs and as tumor‐targeted ADC or peptide‐drug conjugates in combination with ionizing radiation. Both DXd and MMAE induce anti‐tumor immune response that block re‐challenge tumor growth. As monotherapy, DXd is more potent than MMAE at inhibiting tumor formation. In contrast when combined with ionizing radiation at subtherapeutic doses, MMAE but not DXd radiosensitizes resulting in improved tumor control and greater immune activation with MMAE. The differential effects of anti‐tubulin versus topoisomerase I inhibitors when combined with ionizing radiation and immunotherapies can inform and optimize clinical development of ADC based chemo‐radio‐immunotherapy combinations for cancer patients.

## Graphical abstract

Antibody drug conjugates deliver their cytotoxic anti‐tubulin or topoisomerase I inhibitor payloads to tumors through cancer cell receptor targeting. The released drug payloads induce cellular changes that interact with radiotherapy resulting in radiosensitization that improves cancer cell kill and stimulates anti‐tumor immune responses. Such multi‐modal precision oncology therapeutic strategies can overcome tumor resistance and improve cancer patient outcomes.

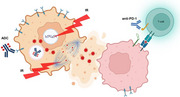

## 1 Introduction

Though potentially curable, locally advanced cancers remain a major cause of patient morbidity and mortality. Their aggressive and infiltrative nature necessitates combinatorial therapeutic approaches with non‐overlapping mechanism of actions and toxicities. To simultaneously attack the identifiable tumor, regional lymph node spread and microscopic metastasis, the chemo‐radiotherapy paradigm was developed over 40 years ago delivering chemotherapy concurrently with radiotherapy.^[^ ^1^ ^]^ Randomized clinical trials across patients with different tumor types have consistently demonstrated concurrent chemo‐radiotherapy improves tumor control and/or patient survival in brain, head and neck, lung, esophagus, cervical, bladder, rectal, and anal cancers compared to monotherapies.^[^ ^2^, ^3^, ^4^, ^5^, ^6^, ^7^, ^8^, ^9^, ^10^ ^]^ There are compelling rationales for combining focal ionizing radiation (IR) with systemically delivered drugs for locally advanced cancers.^[^ ^1^, ^11^ ^]^ Chemotherapy and radiotherapy have independent mechanisms of tumor kill to circumvent resistance from tumor heterogeneity. Radiotherapy directed at visualized tumors and chemotherapy attacking microscopic spread out of the irradiated field provides spatial cooperativity. Finally, certain chemotherapies radiosensitize by increasing IR cancer cell kill. While advances in radiotherapy techniques and molecular targeted therapies are resulting in precision oncology therapies, the chemo‐radiotherapy paradigm continues to rely on non‐targeted cytotoxins that include anti‐metabolites (5‐fluorouracil, gemcitabine), alkylating agents (cisplatin, carboplatin, temozolomide), anti‐mitotics (vincristine, vinblastine, docetaxel, paclitaxel), topoisomerase inhibitors (etoposide) and anti‐tumor antibiotics (mitomycin C).^[^ ^12^, ^13^, ^14^ ^]^ Such chemotherapies increase normal tissue damage in the irradiated field and have systemic toxicities that preclude treatment intensification to further improve tumor control. Synergies from tumor‐directed radiosensitizing cytotoxins offer an opportunity to widen the therapeutic index of chemo‐radiotherapy and potentiate immunotherapies to improve cancer patients survival and quality of life.^[^ ^15^, ^16^ ^]^

Antibody drug conjugates (ADCs) were initially designed to deliver potent cytotoxins to tumors while avoiding normal tissues.^[^ ^17^, ^18^ ^]^ ADCs consist of a chemical linker that couples an antibody to a drug payload that splits the roles of targeting and killing into two distinct molecular tasks. ADC targeting is dictated by the antibody moiety recognizing membrane receptors overexpressed on cancer cells. Membrane receptors validated as ADC targeting beacons for solid tumors include HER‐2, nectin‐4, trop‐2, folate receptor, tissue factor (TF), and c‐Met.^[^ ^19^, ^20^, ^21^, ^22^, ^23^, ^24^ ^]^ ADC tumor kill is mediated by the release of the bound drug payload. ADC payloads are most commonly potent anti‐tubulins (monomethyl auristatin E (MMAE), DM‐1) or topoisomerase I inhibitors (DXd, SN‐38).^[^ ^17^, ^18^ ^]^ In theory, systemically injected ADCs preferentially engage and kill cancer cells while avoiding and sparing normal tissues. However, the clinical experience with ADCs has revealed significant limitations due to toxicities and tumor resistance mediated by cancer cell heterogeneity, ADC linker instability and ADC off target binding.^[^ ^25^, ^26^, ^27^, ^28^ ^]^ To improve the therapeutic index of ADCs, combinatorial use of radiotherapy with ADCs may increase tumor control and/or permit lower effective doses of ADCs given their different mechanisms of cell kill, non‐overlapping toxicities and potential synergies.^[^ ^15^ ^]^ Along with its inherent potent cytotoxicity, MMAE radiosensitized cells by increasing IR induced DNA damage.^[^ ^29^ ^]^ Importantly and for clinical applicability, MMAE‐based ADCs selectively radiosensitized tumors in a receptor restricted manner.^[^ ^30^, ^31^, ^32^, ^33^, ^34^, ^35^ ^]^

In addition to direct cancer cell kill, the tumor immune microenvironment is a major determinant of ultimate tumor control. Within the last decade, immune checkpoint inhibitors have revolutionized cancer treatment.^[^ ^36^, ^37^ ^]^ However, durable response to immunotherapies is limited, which has spurred testing combinatorial strategies including radiotherapy to optimally engage the immune system.^[^ ^38^, ^39^, ^40^ ^]^ IR's unique advantages over chemotherapies include spatial‐temporal controlled dose deposition to irradiated target volumes that results in necrotic cell death and release of tumor antigens to stimulate anti‐tumor immune responses.^[^ ^41^, ^42^ ^]^ Moreover, IR can induce immune‐mediated abscopal effects, i.e., tumor control outside the irradiated field.^[^ ^43^, ^44^, ^45^ ^]^ For immune activation by IR, large 8–20 Gy ablative stereotactic body radiotherapy (SBRT) doses are typically used.^[^ ^42^, ^46^, ^47^ ^]^ In combination with immune checkpoint inhibitors, such larger IR doses given to an isolated tumor target increased immune infiltration and tumor control. However, larger ablative SBRT doses (5‐10 Gy per day) can cause severe complications that include pneumonitis and fatal hemoptysis.^[^ ^48^ ^]^ Given the larger tumor burden of locally advanced cancers, conventionally fractionated radiotherapy (2–3 Gy per day) is given with chemotherapy. Clinical trials testing IR total dose escalation with concurrent chemotherapy has reported increased toxicities with no improvement in overall patient survival.^[^ ^49^ ^]^ ADCs offer a molecularly targeted approach of cytotoxic radiosensitization to widen the therapeutic index of chemo‐radiotherapy by increasing cancer cell kill and stimulate anti‐tumor immune responses that potentiate immunotherapies using clinically established IR doses routinely used in current chemo‐radiotherapy protocols.^[^ ^15^, ^16^ ^]^

Here, we report on the interactions of ADC payloads having different mechanisms of action with radiotherapy and immunotherapy. ADCs furthest along in clinical development or US FDA approved have anti‐tubulin (i.e., MMAE) or topoisomerase I inhibitor (i.e. DXd and SN‐38) payloads but are bound to different targeting antibodies or attached at different drug antibody ratios (DAR).^[^ ^17^, ^20^, ^21^, ^23^, ^50^ ^]^ We focused studies on these two common classes of ADC payloads in combination with IR by testing their ability to radiosensitize and impact tumor control in an immune context as either free drug or as tumor‐targeted antibody/peptide‐drug conjugates by using immune‐competent murine cancer models. ADCs or cell penetrating peptide drug conjugates with MMAE or DXd were synthesized with an identical DAR and targeting moiety to isolate on ADC payload activity with IR. While MMAE was more potent in cell culture, DXd and SN‐38 were more effective in achieving durable tumor control in mouse models. This local tumor control was dependent on the adaptive arm of the immune system, specifically CD8 T‐cells. ADC payloads induced immunogenic cancer cell kill resulting in immunologic memory that prevented tumor growth upon re‐challenge with cancer cells. Interestingly, combining subtherapeutic doses of IR with MMAE but not DXd radiosensitized cancer cell kill. MMAE radiosensitization increased tumor immune stimulation and durable tumor control compared to DXd and IR. The differential radiosensitization of MMAE and DXd provides mechanistic insight to optimally integrate ADCs with radiotherapy and immunotherapies.

## 2 Results

### 2.1 Intrinsic Anti‐Tumor Activity of ADC Payloads

**Figure 1:**
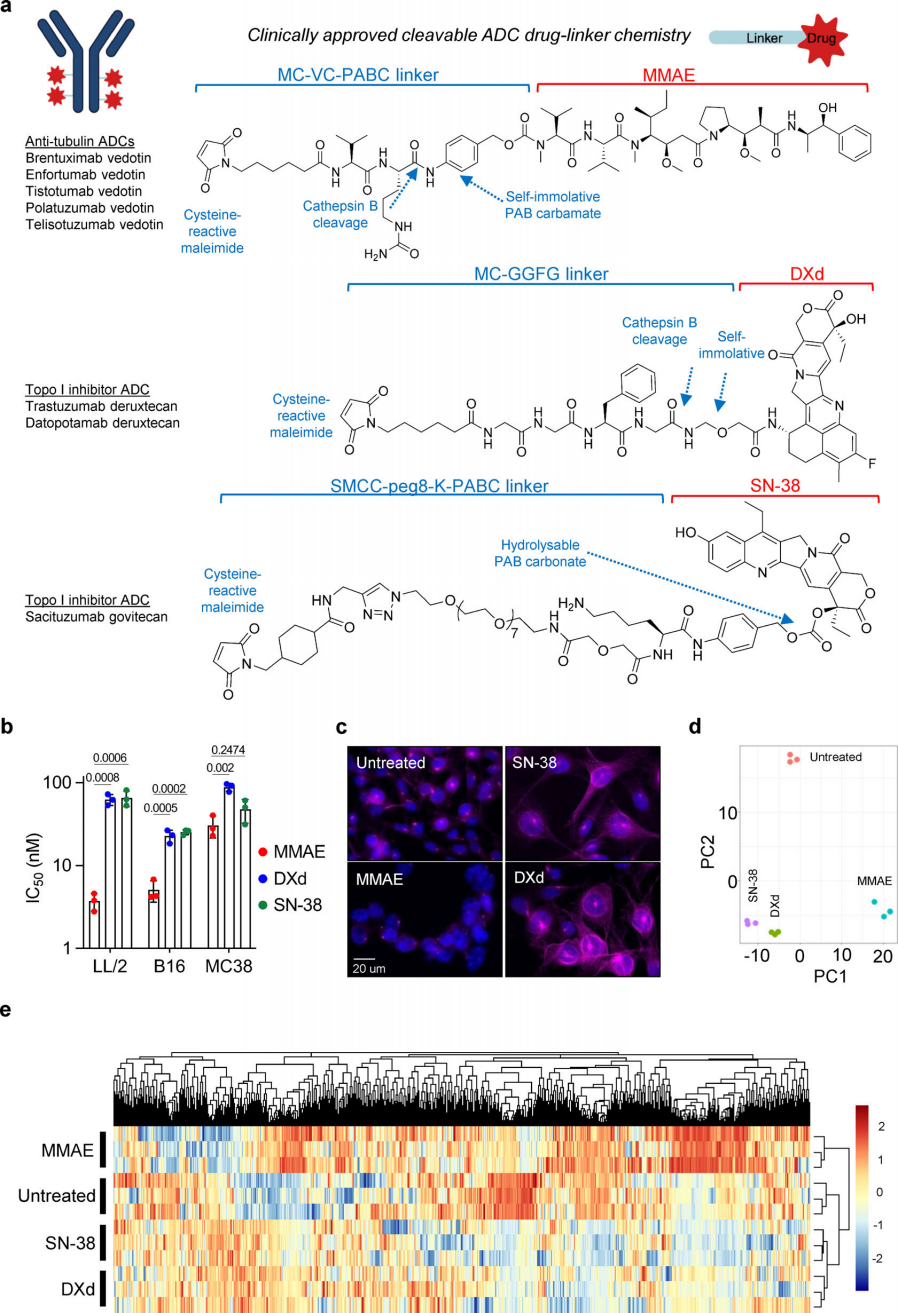
Intrinsic effects of anti‐tubulin and topoisomerase I inhibitor drug payloads in cell culture. a) Schema of drug‐linker chemistry in clinically approved ADCs. Cleavage sites within the linker indicated by dashed blue arrows. b) IC_50_ of MMAE, DXd, and SN‐38 in murine cancer cell lines. Scatter plot with mean of biological replicates, n = 3. Statistical significances calculated using one‐way ANOVA with Tukey's multiple comparisons test. c) Tubulin staining (magenta) of B16 cells treated with 10 nm MMAE, DXd, or SN‐38 for 24 h. Nuclei counterstained with DAPI (blue). d) Principal component analysis plot based on normalized gene expression in B16 cells treated with 10 nm MMAE, DXd or SN‐38 for 24 h, n = 3. RNA expression analyzed using NanoString PanCancer IO 360 Panel. e) Heatmap of gene expression for all samples as described in Figure 1d. The source data used in gene expression analysis is in the Source Data file.

While initially limited to leukemias and lymphomas, clinical efficacy of ADCs in solid tumors has been demonstrated with an expanding number of ADCs with potent cytotoxic anti‐tubulin (i.e., MMAE, DM1) and topoisomerase I inhibitor (i.e., SN‐38, DXd) payloads.^[^ ^20^, ^21^, ^22^, ^23^, ^50^, ^51^ ^]^ For these studies, we focused on ADC payloads with cleavable linker chemistry that results in predictable drug release (**Figure** **1**a).^[^ ^17^ ^]^ MMAE with a MC‐VC‐PABC linker is found in brentuximab vedotin (Adcetris), enfortumab vedotin (Padcev), tisotumab vedotin (Tivdak), polatuzumab vedotin (Polivy), and telisotuzumab vedotin (Emrelis).^[^ ^20^, ^23^, ^24^, ^51^, ^52^ ^]^ DXd with a MC‐GGFG linker is part of trastuzumab deruxtecan (Enhertu) and datopotamab deruxtecan (Datroway).^[^ ^50^, ^53^ ^]^ The alternative topoisomerase I inhibitor SN‐38 is part of sacituzumab govitecan (Trodelvy).^[^ ^21^ ^]^ These three ADC linkers react with IgG hinge cysteines generated by reduction of disulfides through maleimide chemistry to form thiol ethers. The drug payloads are subsequently released by self‐immolative PAB (para‐aminobenzyl) containing linkers that are cleavable by cathepsins after the valine‐citrulline for MMAE and after the phenylalanine‐glycine for DXd. SN‐38 is attached via a hydrolyzable carbonate to the PAB group rather than the more stable carbamate used for MMAE and DXd. The hydrophilic PEG chain in its CL2A linker enables up to eight hydrophobic SN‐38 drugs to be attached that is similar to DXd in trastuzumab deruxtecan.^[^ ^17^ ^]^ In contrast, MMAE containing ADCs are constructed with a DAR of ≈4.

We first evaluated the intrinsic free drug effects of MMAE versus topoisomerase I inhibitor ADC payloads to remove linker, targeting and DAR bias. In cell culture, MMAE was a more potent cytotoxic drug compared to either DXd or SN‐38. The IC_50_ of MMAE was significantly lower than DXd or SN‐38 across all murine cancer cell lines tested (Figure 1b). As expected, treating cells with MMAE but not DXd or SN‐38 destabilized tubulin (Figure 1c; Figure S1, Supporting Information). Globally, principal components analysis (PCA) of MMAE and topoisomerase I inhibitor mediated changes in cancer cell gene expression revealed a distinct clustering of MMAE treated cells compared to control or topoisomerase I inhibitor exposure (Figure 1d).^[^ ^54^, ^55^, ^56^, ^57^ ^]^ Both topoisomerase I inhibitors (i.e., DXd and SN‐38) showed global gene expression changes in cancer cells that were in close proximity to each other. Individual gene expression also showed DXd and SN‐38 treated cells clustered together and distinct from MMAE (Figure 1e). Volcano plot analysis showed MMAE upregulated expression of more genes compared to DXd and SN‐38. Conversely, both topoisomerase I inhibitors decreased the expression of more genes than MMAE (Figure S2a, Supporting Information). Gene Ontology analysis identified MMAE‐induced gene upregulation clustered into immune response and immune cell proliferation pathways (Figure S2b, Supporting Information). In contrast, DXd and SN‐38‐induced gene expression alterations clustered in pathways involved in the cell cycle.

### 2.2 Immune Driven Anti‐Tumor Activity of ADC Payloads

**Figure 2:**
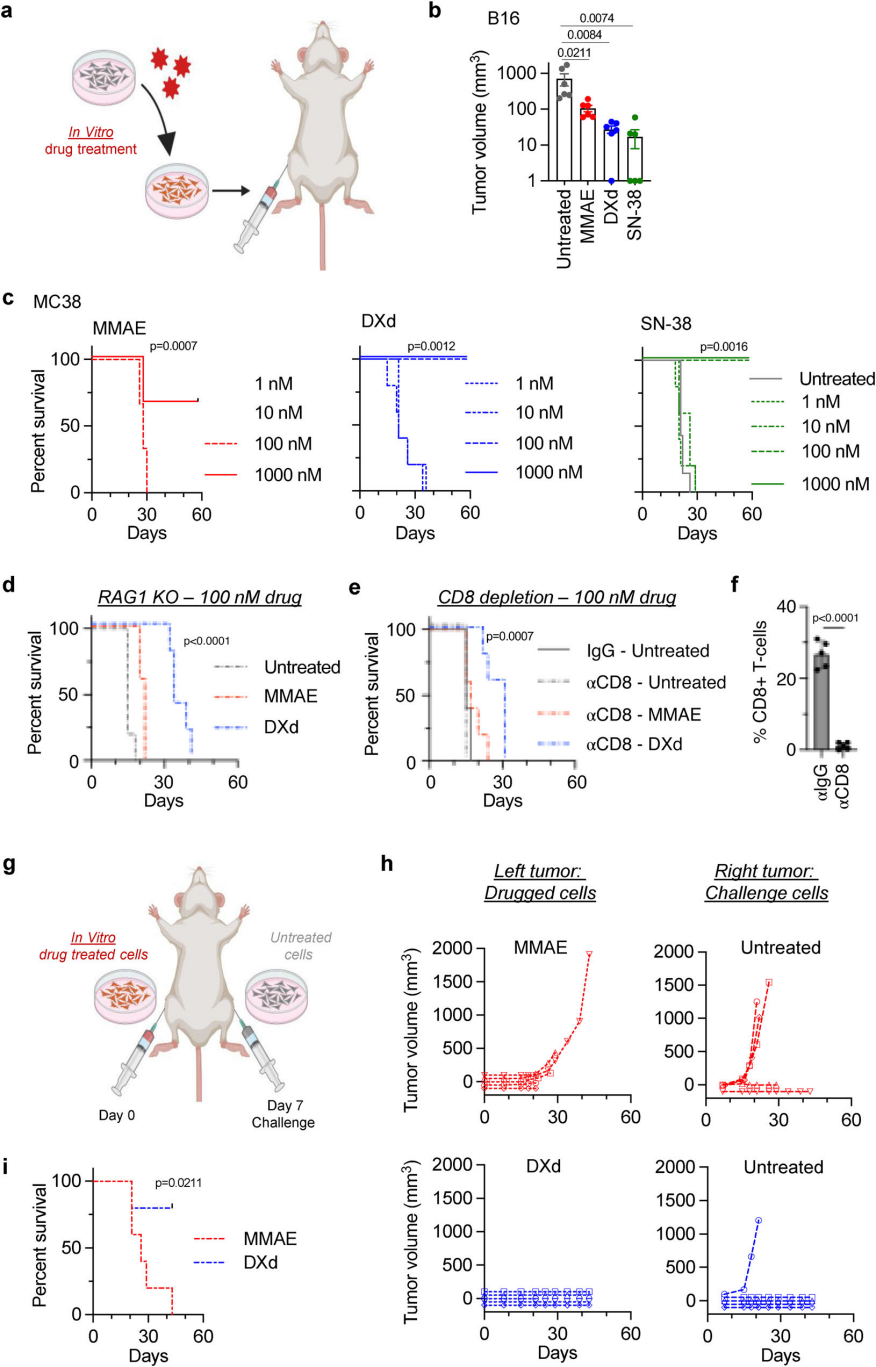
Anti‐tumor activity of auristatin and topoisomerase I inhibitors in immune competent murine models. a) Schematic depiction of in vitro drug treatment followed by in vivo growth assay (created in Biorender.com). Murine cancer cells exposed in cell culture to drug for 24 h were harvested, suspended in drug free media and injected subcutaneously into mice. b) B16 cells exposed to 100 nm MMAE, DXd, or SN‐38 in cell culture and implanted into C57BL/6 mice. Tumor volume scatter plot with mean ± SEM at day 14 post tumor cell implantation, n = 6. Statistical significances calculated using one‐way ANOVA with Tukey's multiple comparisons test. c) MC38 cells exposed to 1, 10, 100, or 1000 nm MMAE, DXd, or SN‐38 in cell culture and implanted into C57BL/6 mice. Mouse survival plotted and statistical significances calculated using Log‐rank (Mantel‐Cox) test, n = 7 (untreated), n = 5 (1, 10 nm), n = 3 (100, 1000 nm). d) MC38 cells exposed to 100 nm MMAE or DXd in cell culture and implanted into RAG1 knockout mice. Mouse survival plotted and statistical significances calculated using Log‐rank (Mantel‐Cox) test, n = 5. e) C57BL/6 mice injected with anti‐CD8 antibody (10 mg kg^−1^) starting 6 days before tumor cell implantation. Drug treated MC38 cells implanted on day 0. Mouse survival plotted and statistical significances calculated using Log‐rank (Mantel‐Cox) test, n = 5. f) Peripheral blood CD8 T‐cell quantification on day 7 from C57BL/6 mice injected with anti‐CD8 antibody as in Figure 2e. g) Schematic depiction of tumor immune challenge assay (created in Biorender.com). Murine cancer cells exposed to drug in cell culture were injected into the left hindlimb. Untreated challenge cells were then injected into the right hindlimb 1 week later. h) MC38 tumor cells treated with 100 nm MMAE (upper panels) or DXd (lower panels) implanted into the left hindlimb followed by untreated challenge M38 cells into contralateral hindlimb. Individual tumor volumes plotted, n = 5. i) Survival curve of mice treated and depicted in Figure 2h n = 5. Statistical significance calculated using Log‐rank (Mantel‐Cox) test.

To determine the tumoricidal effects of ADC payloads in an immune context, we next conducted a series of studies where cancer cells were treated in cell culture with free drugs and then the ability of these in vitro treated cells to form tumors in mice was assessed (**Figure** **2**a). This experimental design was chosen to again eliminate the variabilities and biases introduced by drug conjugation to antibodies and/or peptides. Murine B16 or MC38 cancer cells were treated with MMAE, DXd, or SN‐38 in cell culture. After 24 h of in vitro drug exposure, cells were harvested and viable cells quantitated by trypan blue exclusion. Equivalent numbers of live cells were then resuspended in drug‐free media and implanted subcutaneously into immune‐competent mice. Tumor growth and mouse survival were measured to determine cytotoxicity from the initial 24 h drug pulse. First, B16 cells were treated with 10 or 100 nm of MMAE, DXd, or SN‐38 in cell culture and then implanted into mice. The lower 10 nm dose of MMAE, DXd, or SN‐38 showed no significant tumor growth delay since mouse survival was similar to mice implanted with untreated control cells (Figure S3a, Supporting Information). The higher 100 nm dose resulted in tumor growth delay of drug treated cells compared to implantation of untreated control cells (Figure 2b; Figure S3a, Supporting Information). By day 14, mice implanted with MMAE, DXd, and SN‐38 treated B16 cells all had smaller tumors compared to untreated cell tumor growth. Contrary to MMAE's higher potency in cell culture assays, both topoisomerase I inhibitors (DXd and SN‐38) were more efficacious in the ability to block tumor growth than MMAE that included complete tumor growth inhibition in some mice implanted with DXd or SN‐38 treated cells (Figures 1b and 2b). To substantiate these findings, MC38 cells were exposed to a larger dose range of the drugs in cell culture followed by implantation in mice. As seen with B16 cells, both topoisomerase I inhibitors showed greater cytotoxicity compared to MMAE treated MC38 cells as measured by mouse survival (Figure 2c). For MMAE, MC38 cell culture exposure to 1, 10, or 100 nm of MMAE failed to inhibit tumor formation in all implanted mice, 0% survival by day 30. Only the highest 1000 nm dose of MMAE was able to block MC38 tumor growth resulting in 67% mouse survival. In contrast, mice implanted with MC38 cells exposed to 100 or 1000 nm of DXd or SN‐38 resulted in complete inhibition of tumor growth, 100% survival. A dose response was also seen with the topoisomerase inhibitors. MC38 cells exposed to 1 or 10 nm were ineffective since tumors grew out in all mice and survival similar to mice implanted with untreated MC38 cells.

Since these ADC payloads blocked syngeneic tumor growth in immune‐competent mice, we determined the necessity of anti‐tumor immune responses on local tumor control as opposed to direct cytotoxicity. First, a genetic approach was used where in vitro drug treated cells as above were implanted into RAG1 knockout (KO) mice. RAG1 KO mice have compromised adaptive immunity due to a lack of mature B and T cells.^[^ ^58^ ^]^ Based on the above studies, MC38 cells exposed to an effective 100 nm dose of MMAE or DXd for 24 h in cell culture were implanted in RAG1 KO mice. Tumor growth in immune‐compromised RAG1 KO mice was compared to cells implanted into wild‐type immune‐competent mice. In control untreated MC38 cells, tumors grew slightly faster when implanted in RAG1 KO mice compared to WT mice (Figure S3b, Supporting Information). Although in vitro MMAE exposure increased mouse survival, MMAE treated cells had relative tumor growth curve differences in WT and RAG1 KO mice that paralleled untreated cells (Figure 2d; Figure S3b, Supporting Information). Since both topoisomerase I inhibitors behaved similarly in our above experiments, we focused the remaining studies on DXd in part due to its increasing clinical traction. At 100 nm exposure in cell culture, DXd completely stopped implanted cells from forming tumors resulting in 100% mouse survival in WT mice (Figure 2c; Figure S3, Supporting Information). However, injection of similarly treated DXd cells into RAG1 KO mice resulted in tumor growth in all mice with no long‐term survivors (Figure 2d; Figure S3b, Supporting Information). These results in RAG1 KO mice established adaptive immune responses were necessary for durable control for DXd treated cells. Next, we tested the necessity of the CD8 T‐cell component of adaptive immunity by using a complementary pharmacologic approach to the genetic RAG1 KO mice. WT mice were injected with anti‐CD8 T‐cell depleting antibody and then again implanted with cells treated with 100 nm MMAE or DXd (Figure 2e). Mirroring results in RAG1 KO mice, CD8 T‐cell depletion also completely reversed tumor control of DXd treated MC38 cells seen in WT mice. CD8 T‐cell depletion by antibody treatment was confirmed by flow cytometry of peripheral blood (Figure 2f).

Finally, we tested if ADC payloads induce immunologic memory and distant tumor control using an immunologic challenge assay. MC38 cells again treated with 100 nm MMAE or DXd in cell culture were implanted into the left hindlimb of immune‐competent mice. One week later untreated cells were injected into the contralateral right hindlimb (Figure 2g). While 100 nm MMAE treated cells resulted in tumor growth in the implanted left hindlimb, MMAE did have the capacity to induce immunogenic cell death as seen by rejection of untreated tumor cells injected as a challenge into the contralateral flank with 2 of 5 challenged mice having no tumor growth in the right hindlimb (Figure 2h, upper panels). In agreement with our above data, 100 nm DXd treated cells did not form tumors in the left hindlimb (Figure 2h, lower panels). Importantly, 4 of 5 challenged mice did not have tumors grow out in the right hindlimb that resulted in overall long‐term survival in 80% of mice implanted with both DXd treated cells followed by untreated challenge MC38 cells (Figure 2i). Taken together while MMAE is more potent in cell culture cytotoxicity assays, the topoisomerase I inhibitor ADC payloads DXd and SN‐38 appear more effective in vivo at preventing tumor growth of cells exposed to drug in cell culture. Importantly, the efficacy of ADC payloads is dependent on anti‐tumor immune activation. Engagement of adaptive immunity, specifically CD8+ T‐cells, is necessary for both local (site of treated cell implantation) and distant tumor control (site of untreated challenge cell implantation).

### 2.3 Radiosensitization of ADC Payloads

**Figure 3:**
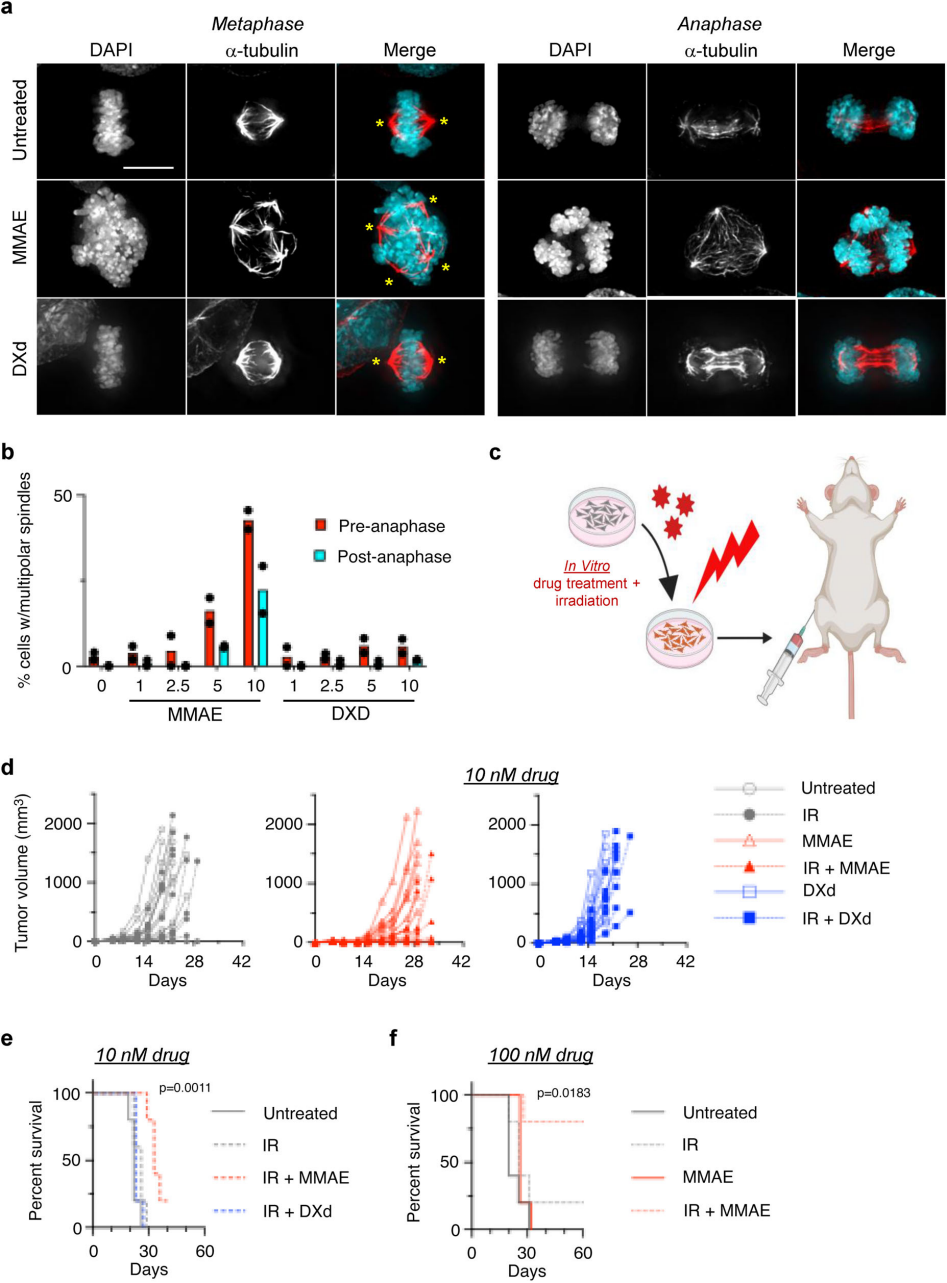
Radiosensitization of auristatin and topoisomerase I inhibitors. a) MC38 cells treated with 5 nm MMAE or DXd for 24 h and stained for α−tubulin, scale bar = 10 µm. Nuclei counterstained with DAPI. In merged images, DAPI is blue, α−tubulin is red, and yellow stars mark spindle poles. b) Quantification of multipolar spindles in MC38 cells treated with a dose range of MMAE or DXd (nm) for 24 h, >50 cells counted in each phase of mitosis/sample. Data plotted as scatter plot, n = 2. c) Schematic depiction of in vitro drug exposure and irradiation followed by in vivo growth assay (created in Biorender.com). Murine cancer cells exposed to drug for 24 h in cell culture followed by IR were harvested, suspended in drug free media and implanted subcutaneously into mice. d) MC38 tumor cells treated with 10 nm MMAE or DXd and 2 Gy IR were implanted into the hindlimb of mice on day 0. Individual tumor volumes plotted, n = 10. e) Survival curve of mice treated and depicted in Figure 3d n = 5. Statistical significance calculated using Log‐rank (Mantel‐Cox) test. f) MC38 cells exposed to 100 nm MMAE for 24 h followed by 2 Gy IR in cell culture were then implanted into C57BL/6 mice. Mouse survival plotted and statistical significances calculated using Log‐rank (Mantel‐Cox) test, n = 5.

We next tested if MMAE or DXd interacted with IR to increase cancer cell kill. Radiotherapy's therapeutic index is in part driven by DNA double strand breaks causing chromosomal aberrations and cell death preferentially in cancer cells over normal tissues.^[^ ^11^ ^]^ Certain drugs can radiosensitize resulting in increased irradiated cancer cell kill and tumor control. Chromosomal instability (CIN) modulates cancer cell sensitivity to IR and is a mechanism for radiosensitization by docetaxel.^[^ ^59^, ^60^ ^]^ Therefore, we determined if ADC payloads differentially induced CIN. MC38 and B16 cells were treated with MMAE or DXd for 24 h and multipolar spindles quantified as a marker of CIN along with mitotic arrest (**Figure** **3**a,b; Figure S4, Supporting Information). Cells were analyzed both pre‐anaphase (prometaphase or metaphase) and post‐anaphase (anaphase and telophase). MMAE increased both pre‐ and post‐anaphase multipolar spindles in a dose dependent manner from 1, 2.5, 5 and 10 nm with a modest increase in mitotic arrest. In contrast, cancer cells treated with DXd did not show appreciable multipolar spindle formation.

To test the ability of MMAE and DXd to radiosensitize in an immune context, we again exposed cells to free drug and irradiation in cell culture prior to implantation into immune‐competent mice to avoid targeting biases introduced by drug conjugation. Subtherapeutic monotherapy drug and IR doses were used to be able to determine interactive cell kill effects of combining drug with IR. MC38 cells were treated in cell culture with 10 nm MMAE or DXd in cell culture for 24 h followed by 2 Gy irradiation (Figure 3c). Following drug exposure and irradiation, cells were harvested, viable cells quantitated by trypan blue exclusion and equivalent cell numbers injected into immune‐competent mice. A drug dose of 10 nm was selected since as monotherapy both MMAE and DXd failed to inhibit tumor growth at 10 nm (Figures 2c and 3d). Cells irradiated with a single 2 Gy fraction showed a modest tumor growth delay compared to implantation of unirradiated tumor cells (Figure 3d left panel). In combination with IR, MMAE radiosensitized as measured by delayed tumor growth and increased mouse survival (Figure 3d middle panel, 3e). In contrast to the interactive effect of IR + MMAE, combining IR with DXd failed to significantly increase tumor control compared to IR alone (Figure 3d right panel, 3e). Importantly, 10 nm MMAE + IR significantly increased mouse survival over IR alone or IR + DXd (Figure 3e). These findings establish MMAE is a more effective radiosensitizer than DXd. However, tumors eventually grew out in all mice treated with 10 nm MMAE + IR as well. To further improve MMAE radiosensitized cancer cell kill for long‐term mouse survival, we then tested 100 nm of MMAE in combination with IR. Again as monotherapy, 100 nm MMAE was ineffective as monotherapy with tumors growing in all mice (Figures 2c and 3f). However, combining 100 nm MMAE + IR significantly improved cell kill resulting in 80% of mice surviving to day 60 after tumor cell implantation (Figure 3f). These results demonstrate MMAE has a distinct radiosensitizing advantage over DXd. This may in part be explained mechanistically by MMAE but not DXd induced formation of multipolar spindles that is a type of CIN resulting in high levels of chromosome missegregation leading to cell death.^[^ ^61^ ^]^

### 2.4 Spatially Targeting ADC Payloads to Tumors in Immune‐Competent Murine Models

**Figure 4:**
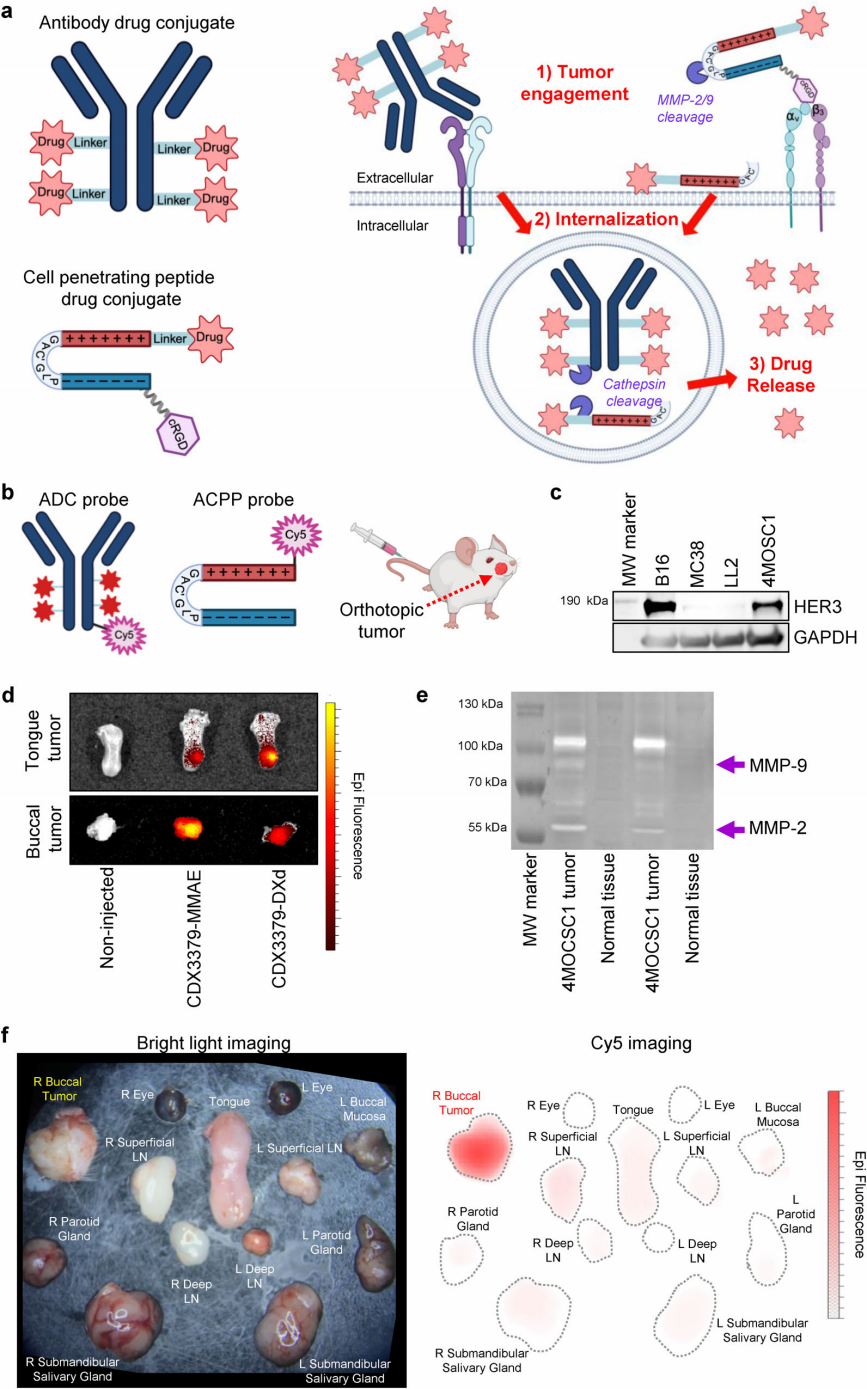
Tumor‐targeted antibody and peptide delivery platforms using identical drug‐linker chemistry. a) Structural representation of ADC with four drugs attached per antibody and ACPP‐drug conjugate with 1 drug attached per cell penetrating peptide using cysteine‐reactive maleimide drug‐linkers. ACPPs consists of a polycationic cell penetrating peptide (+) and autoinhibitory polyanionic peptide (‐) connected by an MMP‐2/9 sensitive peptide linker (PLGC(Me)AG, C(Me) denoted by C’). Sequential steps for ADC and ACPP tumor‐targeting, drug‐conjugate internalization and intracellular drug release. Created in Biorender.com. b) Schematic depiction of Cy5 labeled ADC and ACPP probes. Murine head and neck tumors grown orthotopically in the tongue or cheek followed by i.v. injection of Cy5 labeled ADC or ACPP (created in Biorender.com). c) HER3 expression in murine cancer cell lines. Immunoblot for total HER3 and GAPDH. MW marker ladder in left lane. d) 2.5 nmol Cy5 labeled CDX3379‐MMAE or CDX3379‐DXd i.v. injected into mice with orthotopic cheek or tongue 4MOSC1 tumors, whole tongue or buccal tissue resected and imaged for Cy5 48 h later, pseudocolor Cy5 scale bar on right. e) Gelatin zymography of murine 4MOSC1 tumors and adjacent normal tongue tissue. Molecular weight marker in left lanes. Location of control MMP‐2 and MMP‐9 activity indicated by right arrows. f) Spatial localization of i.v. injected MMP‐2/9 sensitive ACPP in a mouse with 4MOSC1 tumor established in the right buccal mucosa. 10 nmol Cy5 labeled ACPP i.v. injected with tissue harvested 90 min later. Dissected tissues photographed under bright light (left panel) and Cy5 fluorescence imaged (right panel), pseudocolor Cy5 scale bar on far right.

To test the therapeutic efficacy of tumor‐targeted anti‐tubulin and topoisomerase I inhibitors with radiotherapy, we first synthesized complementary MMAE and DXd antibody and peptide‐drug conjugates that removed targeting and DAR bias and could allow us to isolate on the effects of MMAE or DXd and IR within irradiated tumor immune microenvironments. Importantly, the clinical drug‐linker chemistry utilized in ADC construction was used to attach the identical drug‐linker moiety to cell penetrating peptides through the cysteine‐reactive maleimide (Figures 1a and **4**a).^[^ ^34^, ^62^, ^63^ ^]^ Activatable cell penetrating peptides (ACPPs) cloak the polycationic cell penetrating peptide‐drug conjugate within a matrix metalloproteinase (MMP) sensitive scaffold. ACPP architecture consists of a polycationic cell penetrating peptide (9 d‐arginines), a polyanionic autoinhibitory domain (9 d‐glutamic acids) and intervening MMP‐2/9 sensitive peptide linker.^[^ ^64^, ^65^ ^]^ A cRGD attached to the polyanionic moiety pre‐targets ACPPs to tumor overexpressed α_v_β_3_ integrins that are associated with MMP‐2/9.^[^ ^63^ ^]^ Intact, ACPP blocks intracellular entry of its cell penetrating peptide‐drug component. Engaging tumor enriched extracellular MMP‐2 and/or MMP‐9 gelatinase activity cleaves the ACPP to release the polycationic cell penetrating peptide‐drug conjugate that then electrostatically adheres to cell membranes followed by intracellular uptake. ACPPs provide an alternate tumor‐targeted drug delivery platform for ADC payloads that is receptor independent (Figure 4a, right panel). While 4–8 drug molecules are routinely loaded onto antibodies, ACPPs are coupled to only 1 molecule of drug. Importantly, after engagement at the extracellular surface of cell membranes, ADC and ACPPs are both internalized into lysosomes where cathepsins can cleave the drug‐linker to release the attached cytotoxin.

To construct antibody and DAR equivalent ADC tool compounds of MMAE and DXd, the clinically approved drug‐linker combinations (i.e., MC‐VC‐PABC linker for MMAE, MC‐GGFG linker for DXd) were attached to HER3 targeted antibody CDX3379 (Figure 1a; Figures S5 and S6, Supporting Information). CDX3379 has the advantage of recognizing both human and murine isoforms of the HER3 receptor to allow for testing in murine syngeneic tumors grown in immune‐competent mice and interrogating their influence on the tumor immune microenvironment.^[^ ^34^, ^66^, ^67^ ^]^ The DAR of DXd and MMAE loaded ADCs was kept constant at 4 to yield CDX3379‐MMAE and CDX3379‐DXd. Both ADCs were Cy5 labeled allowing in‐vivo tracking (Figure 4b). To confirm tumor targeting of CDX3379‐MMAE and CDX3379‐DXd, mice with orthotopically established syngeneic 4MOSC1 tumors in the tongue or buccal mucosa were intravenously injected with Cy5 labeled CDX3379‐MMAE or CDX3379‐DXd.^[^ ^68^ ^]^ HER3 expression in 4MOSC1 tumors was verified by immunoblotting (Figure 4c; Figure S7b, Supporting Information). Both CDX3379‐MMAE and CDX3379‐DXd localized to orthotopically grown 4MOSC1 tumors (Figure 4d; Figure S7a, Supporting Information). Establishing tumors in the tip of the tongue demonstrated the specificity for tumor‐targeting over adjacent normal tongue tissue for our constructed ADCs. As an alternative tumor‐targeted delivery approach, we synthesized ACPPs conjugated to MMAE or DXd using identical drug‐linkers in clinically approved ADCs (Figure 1a; Figures S8–S13, Supporting Information). Using the same orthotopic 4MOSC1 syngeneic murine tumor model, we confirmed that MMP‐2/9 gelatinase activity was upregulated in 4MOSC1 tumors compared to peri‐tumoral normal tissue (Figure 4e; Figure S7c, Supporting Information). We established ACPP localized selectively to tumors by using a Cy5 labeled ACPP probe (Figure 4b). Cy5 labeled ACPP was i.v. injected into a mouse with an orthotopically established 4MOSC1 tumor in the right buccal mucosa and tissues collected. In tissues dissected from the head and neck region, the ACPP's Cy5 signal preferentially accumulated in the right‐sided buccal 4MOSC1 tumor as opposed to adjacent and regional normal tissues (Figure 4f).

### 2.5 Irradiated Tumor Control and Immune Engagement with Targeted ADC Payloads

**Figure 5:**
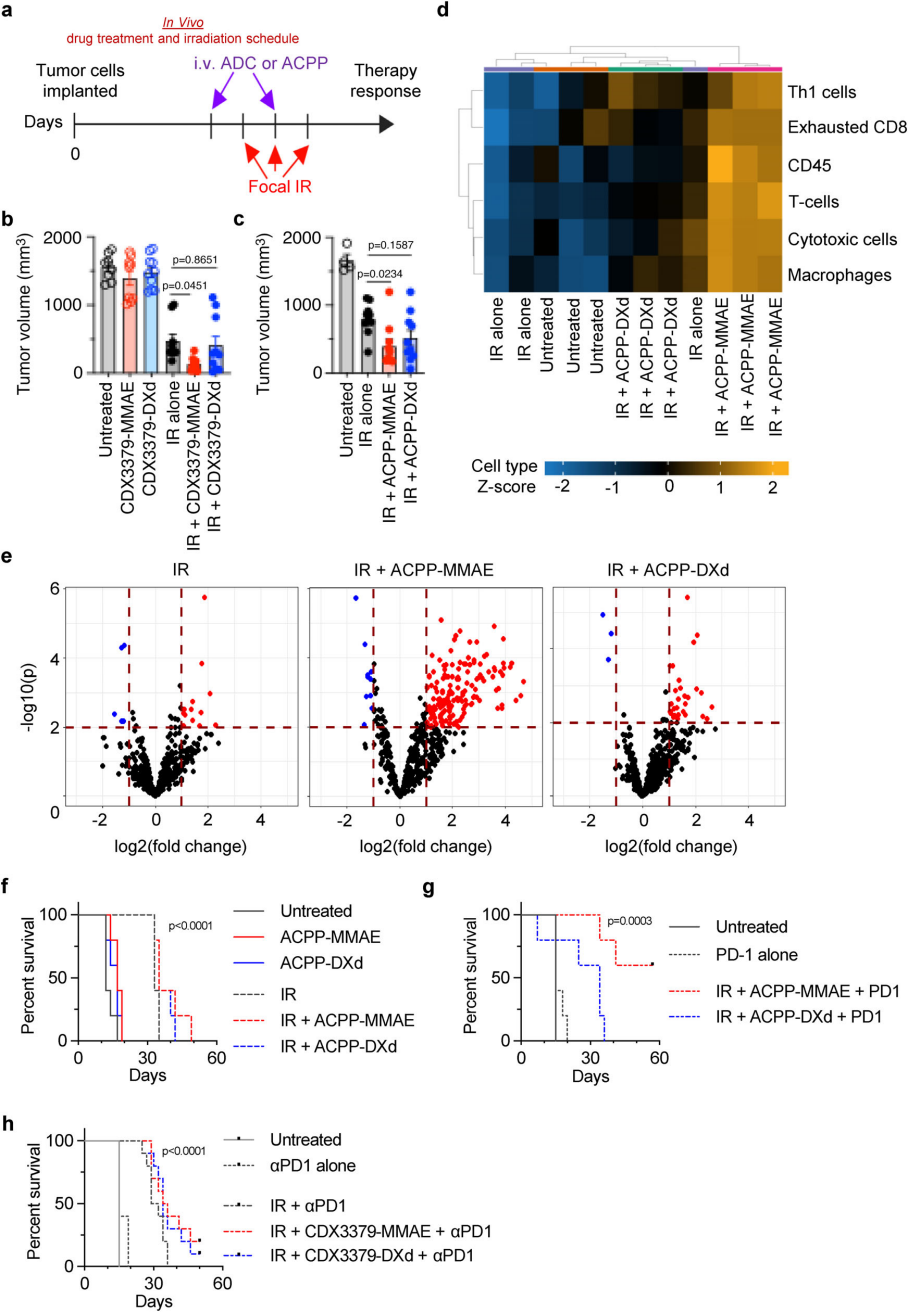
Therapeutic activity of tumor‐targeted ADC payloads with radiotherapy and immunotherapy. a) Treatment scheduling of ADC or ACPP drug conjugates with IR. Tumor cells implanted on day 0. ADC or ACPP drug conjugate given as two doses by i.v. injection and focal IR given to tumor bearing hindlimb as 3 daily fractions starting 1 day after drug treatment initiated. Tumor response and immune system engagement was then assessed. b) Mice with B16 tumors treated with CDX3379‐MMAE or CDX3379‐DXd and IR per Figure 5a starting on day 9 post implantation, n = 8 (untreated), n = 9 (CDX3379‐MMAE, IR, IR+CDX3379‐MMAE), n = 10 (CDX3379‐DXd, IR+CDX3379‐DXd). Tumor volume scatter plot with mean ± SEM at day 18. Statistical significances calculated using one‐way ANOVA with Dunnett's multiple comparisons test. c) Mice with B16 tumors treated with ACPP‐MMAE or ACPP‐DXd and IR per Figure 5a starting on day 9 post implantation, n = 4 (untreated), n = 10 (IR, IR+CDX3379‐MMAE, IR+CDX3379‐DXd). Tumor volume scatter plot with mean ± SEM at day 18. Statistical significances calculated using one‐way ANOVA with Tukey's multiple comparisons test. d) Mice with B16 tumors treated with ACPP‐MMAE or ACPP‐DXd and IR as in Figure 5c. Tumors harvested on day 18 and analyzed using NanoString nCounter PanCancer IO360 panel. Heatmap depicts Z‐score of immune cell type signatures from individual samples, n = 3. Source data in Source Data file. e) Mice with B16 tumors treated with ACPP‐MMAE or ACPP‐DXd and IR as in Figure 5c. Tumors harvested on day 18 and analyzed using NanoString nCounter PanCancer IO 360 panel. Volcano plots for gene expression of IR alone, IR+ ACPP and IR+ACPP DXd versus untreated tumors. Dashed lines represent thresholds for significance in terms of p‐value (horizontal lines) and log2 fold change (vertical lines). Significantly upregulated genes indicated as red dots and significantly downregulated genes indicated by blue dots. Source data in Source Data file. f) Mice with B16 tumors treated with ACPP‐MMAE or ACPP‐DXd and IR per Figure 5a starting on day 3 post implantation. Mouse survival plotted and statistical significance calculated using Log‐rank (Mantel‐Cox) test, n = 5. g) Mice with B16 tumors treated with ACPP‐MMAE or ACPP‐DXd, IR and α‐PD‐1 antibody starting on day 5 post implantation. Mouse survival plotted and statistical significances calculated using Log‐rank (Mantel‐Cox) test, n = 5. h) Mice with B16 tumors treated with CDX3379‐MMAE or CDX3379‐DXd, IR and α‐PD‐1 antibody starting on day 5 post implantation. Mouse survival plotted and statistical significances calculated using Log‐rank (Mantel‐Cox) test, n = 10 (untreated, IR+αPD‐1), n = 5 (αPD‐1 alone), n = 8 (IR+CDX3379‐MMAE+αPD‐1), n = 9 (IR+CDX3379‐DXd+αPD‐1).

Finally, we tested the efficacy tumor‐targeted MMAE or DXd in combination with IR on tumor control and potentiation of immunotherapy. Immune‐competent mice with established syngeneic B16 tumors were treated with systemically administered tumor‐targeted MMAE or DXd delivered as antibody drug conjugate (CDX3379‐MMAE, CDX3379‐DXd) or cell penetrating peptide drug conjugate (ACPP‐MMAE, ACPP‐DXd) and focal tumor irradiation scheduled as shown in **Figure** **5**a. Murine B16 tumors express HER3 and MMP 2/9 making them targetable by anti‐HER3 antibody CDX3379 or ACPPs (Figure 4c).^[^ ^69^ ^]^ For ACPP or ADC, two i.v. injected doses were given 48 h apart from each other. Each ADC injection dose was 2.5 nmol (19 mg kg^−1^) and each ACPP injection was 10 nmol (2.9 mg kg^−1^). Since our ADCs were constructed with four drug molecules/antibody (DAR = 4) and ACPPs synthesized with one drug molecule/cell penetrating peptide, the total drug injected from the two doses of 2.5 nmol of ADC or two doses of 10 nmol ACPP resulted in an equivalent of 20 nmol total drug payload administered. Our previous work has shown that such a dosing regimen of ACPP‐MMAE or CDX3379‐MMAE resulted in equivalent tumor drug concentrations.^[^ ^34^, ^69^ ^]^ Focal IR to the tumor bearing hindlimb was started the day after first i.v. injection of ADC or ACPP. IR was delivered daily for three consecutive days to mimic clinically fractionated radiotherapy and allow for interactions with MMAE or DXd including radiosensitization. In short‐term studies, CDX3379‐MMAE or CDX3379‐DXd as monotherapy had minimal effects on tumor regression by day 18 post tumor cell implantation (Figure 5b). As expected, IR monotherapy was effective at slowing tumor growth. Interestingly, mice treated with IR in combination with CDX3379‐MMAE had significantly smaller tumors compare to IR alone or ADC alone (Figure 5b). In contrast, mice treated with both IR and CDX3379‐DXd had tumors that were not significantly different in size than IR alone. Using the alternative ACPP drug delivery platform with IR produced similar results to ADCs + IR (Figure 5b,c). IR monotherapy again slowed tumor growth. As seen with IR + CDX3379‐MMAE, IR combined with ACPP‐MMAE had significantly smaller tumors compared to IR alone. While IR + ACPP‐DXd treated mice also had smaller tumors on average than IR alone, this did not reach statistical significance. Importantly, two different drug delivery targeting platforms (receptor‐dependent ADC and receptor‐independent ACPP) for MMAE and DXd showed similar effects on irradiated tumor control.

Since contrasting radiosensitization potential was seen with MMAE and DXd, we then investigated if MMAE or DXd combined with IR differentially stimulated tumor immune infiltration. Mice were treated as above with CDX3379‐MMAE or CDX3379‐DXd and IR (Figure 5a). Alterations to specific immune cell populations as a percentage of CD45+ immune cells were analyzed by flow cytometry (Figure S14a, Supporting Information). For total CD8+ T cells, CDX3379‐MMAE and CDX3379‐DXd did not alter tumor immune infiltration (Figure S14b, Supporting Information). Irradiation of tumors increased total CD8+ T cells that was minimally changed with MMAE or DXd. In contrast, the memory CD8+ T‐cell subset (CD44+, CD62L+) increased in irradiated tumors treated with CDX3379‐MMAE or CDX3379‐DXd. Immune suppressive Tregs counteract anti‐tumor responses within the tumor immune microenvironment. While a smaller proportion of immune cells, IR induced upregulation of Tregs was further exacerbated by the addition of MMAE or DXd. Using the alternative ACPP drug delivery system, we observed similar trends in tumors harvested from mice treated with IR + ACPP‐MMAE or IR + ACPP‐DXd compared to untreated or IR alone on T‐cells and Tregs (Figure S14c, Supporting Information). These results highlight ADC payloads can have counteracting effects within the irradiated tumor immune microenvironment. To more comprehensively investigate tumor‐immune alterations in an unbiased manner, NanoString analysis was utilized. Tumor bearing mice were treated with IR alone or in combination with ACPP conjugated to MMAE or DXd. Clustering by immune cell types revealed an increase in CD45+ immune cells, macrophages, and cytotoxic cells in tumors from mice treated with IR + ACPP‐MMAE compared to untreated, IR alone, or IR + ACPP‐DXd mice (Figure 5d). At the individual gene expression level, IR + ACPP‐MMAE resulted in a striking increase in the number of genes upregulated compared to untreated and tumors treated with IR alone or IR + ACPP‐DXd tumors (Figure 5e; Figure S15a, Supporting Information). By gene Ontology pathway analysis, MMAE or DXd differentially upregulated immune pathways within the irradiated tumor immune microenvironment (Figure S15b, Supporting Information). The top 3 pathways upregulated by IR alone involved chemokine responses, cytokine signaling, and leukocyte migration. IR + ACPP‐DXd upregulated genes involved in infectious response, cytokine signaling and chemokine production. In contrast, IR + ACPP‐MMAE upregulated pathways involved in immune effector processes, leukocyte proliferation, and immunity.

For therapeutic efficacy, we tested for long‐term durable tumor control of targeted MMAE or DXd and IR. Mice treated with IR + ACPP‐MMAE had the longest survival (Figure 5f). However, tumors eventually grew out in all mice and no mice survived past 49 days post tumor cell implant. Given the immune modulatory effects of MMAE and DXd when combined with IR including exhausted CD8 T‐cells, integration with immunotherapies could provide an orthogonal approach for tumor control (Figure 5d). Therefore, we tested if immune checkpoint inhibitors improved irradiated tumor control when combined with tumor‐targeted MMAE or DXd. Based on optimization studies, anti‐PD‐1 immune checkpoint therapy was started one week after initiation of IR and tumor‐targeted MMAE or DXd. Anti‐PD‐1 was intraperitoneally (i.p.) injected on day 12, 15, and 19 post cancer cell implantation. To reduce drug delivery bias, we tested if anti‐PD‐1 therapy improved irradiated tumor control in combination with MMAE or DXd irrespective of the coupled carrier vehicle delivering drug, i.e., cell penetrating peptide (ACPP) or antibody (ADC). Focusing on just varying drug payload (i.e., MMAE vs DXd), IR + ACPP‐MMAE followed by anti‐PD‐1 therapy improved mouse survival when directly compared to IR + ACPP‐DXd followed by anti‐PD‐1 therapy (Figure 5g). Importantly, such a trimodal strategy of chemo‐radio‐immunotherapy showed similar results with the ADC drug delivery platform. ADCs with MMAE or DXd given in combination with IR followed by anti‐PD‐1 antibody improved durable tumor control compared to IR + anti‐PD‐1 (Figure 5h). While not as dramatic, the MMAE payload consistently showed improved tumor control irrespective of drug delivery platform when combined with IR when directly compared to IR + DXd. From a safety point, the combination of tumor‐targeted MMAE or DXd delivered as an ADC or ACPP conjugate with IR and an immune checkpoint inhibitor was tolerated as assessed by murine body weight (Figure S16, Supporting Information).

## 3 Discussion

Concurrent chemo‐radiotherapy transformed the treatment of locally advanced cancer therapy over 40 years ago.^[^ ^1^ ^]^ While progress in radiotherapy delivery and immunotherapy discoveries have moved toward precision oncology, chemotherapies given with radiotherapy remain the same non‐targeted cytotoxins from decades old chemo‐radiotherapy trials. ADCs offer a solution for molecularly targeting cytotoxic drugs to irradiated tumors. Our and other previous work focused on anti‐tubulin based radiosensitization, however, ADCs with topoisomerase I inhibitors are gaining clinical traction and expanding the translational potential of ADC based chemo‐radiotherapy multimodal therapy for cancer patients.^[^ ^29^, ^30^, ^31^, ^32^, ^33^, ^34^, ^35^, ^70^ ^]^ The current studies investigated the interactions of anti‐tubulin (MMAE) versus topoisomerase I inhibitor (DXd, SN‐38) ADC payloads with IR in an immune context. To remove targeting and DAR bias, we evaluated free ADC payloads and then attached these MMAE and DXd to the same antibody (anti‐HER3 specific CDX3379) or cell penetrating peptides (ACPP). ADCs were synthesized at equivalent DAR of 4:1 or drug:cell penetrating peptide ratio of 1:1. However, MMAE and DXd were attached with different linker chemistries. For clinical relevance, we used clinically validated drug‐linker chemistries from approved ADCs, MC‐VC‐PABC linker for MMAE and MC‐GGFG linker for DXd.^[^ ^17^ ^]^ A strength of these studies is that the advantage of MMAE over DXd with regards to tumor radiosensitization was seen not only as free drug but also irrespective of tumor‐targeting delivery system indicating radiosensitization to be an inherent property of MMAE and may be translatable across MMAE‐based antibody and peptide drug conjugates being developed.

Curiously, while MMAE was more potent in cell culture, topoisomerase I inhibitors DXd and SN‐38 were more effective at cancer cell kill in immune‐competent mice. This may be in part be explained by the fact that the immune system played a critical role in the local tumor control of DXd through the adaptive arm of the immune system, specifically CD8 T‐cells. Using immunologic challenge assays, both MMAE and DXd cell kill induced immunologic memory that attacked untreated tumor cells. Interestingly, while the topoisomerase I inhibitors were more effective as monotherapy, IR + MMAE outperformed IR + DXd in syngeneic mouse models. Testing for interactions with IR, we found MMAE radiosensitized more effectively than DXd. This result is in line with prior work demonstrating MMAE radiosensitized in cell culture and immune‐deficient human tumor xenograft models.^[^ ^29^, ^30^, ^31^, ^33^, ^34^ ^]^ Mechanistically, MMAE induced chromosomal instability that was not seen with DXd. CIN is known to stimulate anti‐tumor immunity, thus, MMAE‐induced CIN also may play a role in the enhanced anti‐tumor immune responses observed when MMAE was combined with IR.^[^ ^59^, ^60^ ^]^ Within the tumor immune microenvironment, IR combine with ACPP‐MMAE showed distinct increases in immune pathways involved in immune effector processes, leukocyte/lymphocyte proliferation, and adaptive immune responses that was not found with DXd. While not as an effective radiosensitizer as MMAE, the increased in vivo potency of DXd may result in additive effects in combinations with radiotherapy and immunotherapy. Given the immunomodulatory effects of DXd, MMAE, and IR, IR delivered together with tumor‐targeted MMAE or DXd improved tumor control in combination with immune checkpoint inhibitors.^[^ ^34^, ^69^, ^71^, ^72^ ^]^

A motivation for these studies stems from the clinical limitations of ADCs in cancer patients that include tumor heterogeneity, resistance, dose limiting toxicities (both on and off target) all of which may influence efficacy depending on individual antibody and DAR characteristics of a particular ADC.^[^ ^18^, ^26^ ^]^ As a solution to this clinical problem, integrating ADCs with radiotherapy can leverage additive and/or synergistic radiosensitization mechanisms of tumor kill to improve the therapeutic index of concurrently delivered ADCs and radiotherapy.^[^ ^15^ ^]^ Moreover, ADC radiosensitization induced immunogenic death resulting in increased tumor immune infiltration can be therapeutically exploited with the use of immune checkpoint inhibitors. Such a multi‐pronged trimodal ADC‐radio‐immunotherapy attack may overcome tumor heterogeneity and resistance that improve outcomes for cancer patients. Our CDX3379 based ADCs target HER3 while current clinically approved ADCs for solid tumor include those that target HER2, Trop‐2, Nectin 4, TF, and c‐Met.^[^ ^19^, ^20^, ^21^, ^23^, ^24^, ^50^, ^53^ ^]^ Translationally, many patient cancers treated with chemo‐radiotherapy express actionable receptors by these approved MMAE and/or DXd ADCs providing opportunities for clinically evaluating ADC based chemo‐radiotherapy with immunotherapies.^[^ ^15^ ^]^ Clinical evidence is emerging on the use of ADCs with radiotherapy including case reports of exceptional tumor responses seen with MMAE containing ADCs. In patients with cutaneous T‐cell lymphoma, brentuximab vedotin given concurrent with low‐dose total skin irradiation achieved near complete responses.^[^ ^73^ ^]^ In patients with metastatic urothelial cancer, enfortumab vedotin combined with radiotherapy resulted in tumor control.^[^ ^74^ ^]^ Given the growing clinical use of ADCs, safety data is also emerging on the tolerability of ADCs given with radiotherapy especially for HER2 targeted ADCs used in breast cancer patients. Both trastuzumab emtansine and trastuzumab deruxtecan have acceptable safety profiles when given with radiotherapy in breast cancer patients.^[^ ^75^, ^76^ ^]^ However, there appears to be an increased risk of brain radionecrosis when the anti‐tubulin ADC trastuzumab emtansine is given with cranial irradiation.^[^ ^16^, ^75^ ^]^ This may in part tie into anti‐tubulins being a more effective radiosensitizers than topoisomerase I inhibitors. While in early days, clinical trials are also now exploring the use of concurrent ADCs with radiotherapy.^[^ ^77^, ^78^ ^]^ Given the approval of enfortumab vedotin in bladder cancer and the routine use of cisplatin‐based chemo‐radiotherapy as a bladder preserving treatment, clinical trials are testing enfortumab vedotin with radiotherapy, (NCT06394570, NCT06434350) and also trimodal enfortumab vedotin, radiotherapy with pembrolizumab (NCT06470282). Though ADCs offer a precision oncology‐based approach to concurrent chemo‐radiotherapy, variation in receptor expression between patients may require personalization of ADC choice as opposed to class solutions offered by conventional chemotherapeutics. Trial designs such as that being tested in The ADC MATCH Screening and Treatment Trial (NCT06311214) may need to be utilized. In this trial, the choice of ADC used (i.e., sacituzumab govitecan, enfortumab vedotin, or trastuzumab deruxtecan) is matched based on expression of Trop‐2, nectin‐4, or HER2. In summary, our pre‐clinical studies testing IR with anti‐tubulin and topoisomerase I inhibitor ADC payloads as free drugs, peptide drug conjugates, and ADCs support continuing to test them into the historically successful chemo‐radiotherapy paradigm to progress from non‐targeted cytotoxic chemotherapies toward ADC directed chemo‐radio‐immunotherapy combinations.

## 4 Experimental Section

### Cells and Reagents

Murine LL2 (CRL‐1642) and B16 (CRL‐6475) cell lines were obtained from American Type Culture Collection. Murine MC38 (ENH204‐FP) cell line was obtained from Kerafast. Murine head and neck 4MOSC1 cancer cells were generated in Dr. Gutkind's laboratory and previously characterized.^[^ ^68^ ^]^ LL2 and B16 cells were cultured in DMEM (Gibco 11960044) supplemented with 10% FBS (Omega Scientific FB02). MC38 cells were cultured in DMEM supplemented with 10% FBS, 1 mm sodium pyruvate (Gibco 11360070), 1% non‐essential amino acids (Gibco 11140050), and 10 mm HEPES (Gibco 15630080). 4MOSC1 were grown on collagen coated plates in Defined Keratinocyte SFM (serum free media) (Gibco 10744019) supplemented with 5ng mL^−1^ Mouse EGF Recombinant Protein (Thermo Fisher PMG8044), 5×10^−11^ m cholera toxin (Millipore Sigma C8052), and 1X Antibiotic Antimycotic Solution (Millipore Sigma A5955). On initial receipt, cell lines were expanded, and low passage stocks cryopreserved. Cells were regularly tested for mycoplasma by PCR. MMAE (Selleck Chemicals S7721), DXd (MedChem Express HY‐13631D), and SN‐38 (MedChem Express HY‐13704) were dissolved in DMSO for use in cell culture.

### Cytotoxicity Assay

Murine cancer cell lines were plated in 96‐well plates and exposed to a drug concentration range of 0–1000 nm for 72 h. Alamar Blue (Invitrogen DAL10025) was added to the cells and allowed to incubate for 2–4 h at 37 °C. Plates were analyzed using a plate reader with fluorescence measured at 560 nm. Fractional survival was normalized to untreated control cell fluorescent values.

### Tubulin Staining

Cells were rinsed with warm (37 °C) microtubule stabilizing buffer (MTSB:100 mm 1,4‐piperazinediethanesulfonic acid, pH 6.9, 30% glycerol, 1 mm ethylene glycol tetraacetic acid, and 1 mm magnesium sulfate) then permeabilized in warm MTSB containing 0.5% Triton X‐100 for 5 min then rinsed with warm MTSB followed by fixation in 4% paraformaldehyde diluted in MTSB for 10 min at room temperature. Cells were blocked with Triton Block (0.2 m Glycine, 2.5% fetal bovine serum, 0.1% Triton X‐100 in phosphate buffered saline) for 1 h at room temperature then incubated in rat anti‐tubulin (YL1/2, Bio‐Rad, MCA77G) diluted 1:1000 in Triton Block for 1 h. Coverslips were washed 3×5 min with 0.1% Triton X‐100 in PBS then incubated with donkey anti‐rat IgG secondary antibody, Alexa Fluor 594 (Invitrogen, A‐21209) diluted 1:200 in Triton Block for 45 min at room temperature in the dark. Coverslips were washed 3 × 5 min with 0.1% Triton X‐100 in PBS then incubated with 0.5 µg mL^−1^ DAPI in PBS for 2 min then rinsed once with PBS and mounted on a glass slide with ProLong Diamond Antifade mountant (Invitrogen, P36965). Images were acquired on a Nikon Eclipse Ti2‐E inverted fluorescence microscope using a Hamamatsu ORCA‐FusionBT back‐thinned camera or a Hamamatsu Orca Flash 4.0 camera and a 100x/1.4 numerical aperture (NA) oil objective. Images are maximum projections of 0.2‐µm z‐stacks deconvolved using Nikon Elements software.

### Synthesis of Cy5 Labeled Antibody Drug Conjugates

A solution (50 mg mL^−1^) of CDX3379 (kindly provided by Celldex) was diluted (x10–20 fold) with water and solutions of sodium bicine buffer (1 m pH 8.3) and sodium diethylenetriaminepentaacetic acid (100 mm pH 7) added to give final concentrations of 100 and 1 mm, respectively. The concentration of antibody was determined by absorbance at 280 nm using an extinction coefficient of 225 750 m ^−1^cm^−1^. Following reduction with four equivalents of tris(carboxyethyl)phosphine hydrochloride (from a 10 mm solution in water) at 37 °C for 2h, the solution was added to four equivalents of maleimide‐linker‐drug (10 mm solution in DMSO that had been prediluted with an equal volume of water immediately before addition) and mixed rapidly by pipet. After 30 min at room temperature, the reaction mix was added to two equivalents of Cy5‐maleimide (10 mm solution in DMSO; prediluted with an equal volume of water immediately before addition) and after a further 30 min, gel‐filtered (Sephadex G25, 1.0 g) eluting with PBS, collecting the first blue colored band. Following centrifugal concentration (Centricon 30 kD MWCO), the concentrations of antibody, drug (for deruxtecan) and Cy5 were determined by absorbance using extinction coefficients of 225 750 m ^−1^cm^−1^ (CDX3379) at 280 nm and 12 500 m ^−1^cm^−1^ and 250 000 m ^−1^cm^−1^ at 280 and 650 nm respectively for Cy5. For Deruxtecan‐Cy5 conjugates, additional extinction coefficients of 18 100 and 5000 m ^−1^cm^−1^ at 363 and 280 nm, respectively for Deruxtecan and 7000 m ^−1^cm^−1^ at 363 nm for Cy5 were used. The solution was diluted with PBS to a final antibody concentration of 100 µm. Drug loading was measured by denaturing reverse‐phase HPLC or by absorbance. ADCs were prepared immediately prior to biological experiments. Synthesis specific details for MMAE and DXd conjugation follow.

*CDX3379‐MMAE Specific Methodology*: Following the general method using maleimidocaproyl‐valine‐citrulline‐PABC‐MMAE (Sigma–Aldrich, 646502‐53‐6). Yield, 90%. Drug loading = 3.4–3.9 as determined by HPLC, (Agilent PLRP‐S 1000 Å 8 µm 2.1 × 50 mm column at 90 °C; 20–55% gradient of acetonitrile‐water‐0.05% TFA in 14 min) of the reaction mix prior to addition of Cy5 maleimide, following reduction of any remaining intersubunit disulfides with 50 mm DTT for 30 min. Peaks corresponding to light or heavy chains with 0–3 MMAE were identified by retention times and electro‐spray mass spectroscopy as before and peak areas at 280 nm were integrated and weighted to calculate the drug loading. Modified light chain (L1) and unmodified H chain (H0) were poorly resolved so determining MMAE loading is less accurate, but no free MC‐VC‐PABC‐MMAE was detected by HPLC following reaction indicating complete reaction with four equivalents of drug linker.

*CDX3379‐DXd Specific Methodology*: Following the general method using maleimidocaproyl‐ glycine‐ glycine‐phenylalanine‐glycine‐DXd (MedChemExpress HY‐13631E). Yield, 88–94%. Drug loading = 3.0–3.8, determined by absorbance (as modified heavy chains were poorly resolved on HPLC) using extinction coefficients of 225 750 m ^−1^cm^−1^ (for CDX3379) at 280 nm, and 12 500, 7000, and 250 000 m ^−1^cm^−1^ at 280, 363, and 650 nm, respectively, for Cy5, and 18 100 and 5000 m ^−1^cm^−1^ at 363 and 280 nm, respectively, for MC‐GGFG‐DXd. As the absorbance of conjugated MC‐GGFG‐DXd differed from unconjugated linker by their relative values at the 360 and 380 nm peaks with additional red‐shifted absorbance at ≈420 nm, this value for drug loading is approximate, but no free MC‐GGFG‐DXd peaks were detected by HPLC after reaction, indicating complete reaction with four equivalents of drug linker.

### Synthesis of ACPP‐Drug Conjugates

Peg8‐ACPP, H_2_N‐peg8‐e_9_‐oPLGC(Me)AG‐r_9_‐c‐CONH_2_. 10·TFA salt, was prepared using regular solid phase Fmoc peptide synthesis and purified by reverse‐phase HPLC, where lower case letters refer to D‐amino acids, peg8 refers to H_2_N‐PEG8‐propionic acid, o‐denotes 5‐amino‐3‐oxopentanoyl (a short hydrophilic spacer), C(Me) denotes for S‐methylcysteine and the CONH_2_ indicates C‐terminal amide). Peptide was dissolved in dry DMSO (at 10–20 mm) and mixed with drug‐linker‐maleimide (1 equiv as a 10 mm solution in DMSO) and N‐methylmorpholine (10 equiv) and kept at room temperature for 30–60 min. Following confirmation of complete reaction of peptide by reverse phase HPLC, a solution of 6‐maleimidohexanoic acid N‐hydroxysuccinimide ester (Sigma–Aldrich, 63177; 1 equiv. as a freshly prepared 100 mm solution in DMSO) was added to the reaction mixture and kept at room temperature for 4–5 days until HPLC indicated reaction was complete. Cyclo(RGD)fC (Vivitide, PCI‐3686‐PI), 1.2 equiv as a 25 mm solution in DMSO) was added and mixed. HPLC indicated complete reaction after 30 min to yield the final product, and the reaction was quenched with acetic acid, separated by HPLC and lyophilized to give the product. Following storage as a powder at −20 °C, stock solutions were prepared in water at 1 mm concentration and stored frozen at −20 °C. Synthesis specific details for MMAE and DXd conjugation follow.

*ACPP‐MMAE Specific Methodology*: Prepared using MC‐VC‐PABC‐MMAE (VC‐MMAE, Sigma–Aldrich, 646502‐53‐6) to give a white powder, yield 52%. Purity (99%). HRMS (ESI‐TOF) calculated *m*_average_ for C_249_H_409_N_75_O_79_S_3_ 5813.57 Da, found 5812.9. Calculated *m*_monoisotopic_ 5809.9455 Da, found 5809.912.

*ACPP‐DXd Specific Methodology*: Prepared using MC‐GGFG‐DXd (MedChemExpress, HY13631E) to give a pale‐yellow powder, yield 50%. Purity (99%). HRMS (ESI‐TOF) calculated *m*_average_ for C_233_H_360_FN_73_O_77_S_3_ 5530.9964 Da, found 5530.6. Calculated *m*_monoisotopic_ 5527.5645 Da, found 5527.543.

### In Vivo Subcutaneous Tumor Studies

All animal work was done in compliance with the University of California San Diego Institutional Animal Care and Use Committee, protocol# S15290. Mice were housed in individually ventilated and micro‐isolator cages supplied with acidified water and 5053 irradiated PicoLab Rodent Diet 20 feed. Temperature for laboratory mice in housing was 18–23 °C with humidity 40–60%. Housing room was maintained on a 12 h light/dark cycle. For in vitro cell culture drug exposure and irradiation followed by mouse implantation, B16 or MC38 cells were exposed to MMAE, DXd or SN‐38 for 24 h and then irradiated in cell culture. Cells were collected, resuspended in 100 µL of drug free 1:1 Growth Factor Reduced Matrigel (Corning 354230), and PBS solution then subcutaneously injected into hindlimbs of 6‐week‐old female C57BL/6 WT or RAG1 KO mice (Jackson Labs). For CD8 depletion studies, mice were intraperitoneally (i.p.) injected with anti‐CD8 (BioXCell #BE0117) at 10 mg kg^−1^ on days ‐6, ‐5 and ‐4 tumor cell implant and then weekly starting on day after tumor cells were injected or control antibody. For targeted drug delivery experiments, mice were intravenously injected with CDX3379‐MMAE, CDX3379‐DXd, ACPP‐MMAE or ACPP‐DXd as indicated in Figure Legends and Results given as two doses separated by 48 h. Fractionated 5 Gy IR was delivered focally to the tumor bearing hindlimbs for three consecutive days starting on day after initial ADC/ACPP injection using a PXI X‐RAD 225 XL irradiator at maximal dose rate of 3 Gy min^−1^ with a beam conditioning copper filter (Precision X‐ray Irradiation). For anti‐PD‐1 therapy, mice were i.p. injected with 200 µg anti‐PD‐1 antibody (BioXCell #BE0033) or control antibody on days 12, 15 and 19 after tumor cell implantation. Tumor volumes were measured and calculated using the formula as 1/2 x Length x Width^2^. Mice were sacrificed when tumor volume reached > 1500 mm^3^, maximal tumor volume approved on the protocol for subcutaneous tumors.

### Imaging of Cy5 Labeled ADC and ACPP

For orthotopic tumors, 6‐week‐old female C57BL/6 mice were injected with 4MOSC1 cells into the right buccal mucosa (500 000 cells) or tongue (150 000 cells). One week later, mice were intravenously i.v. injected with 2.5 nmol Cy5 labeled ADC or 10 nmol Cy5 labeled ACPP. Oral cavity tumor and normal tissues were dissected out 48 h post ADC injection or 90 min post ACPP injection, bright light photographed, and Cy5 fluorescence measured using IVIS 200 (Xenogen).

### Gelatin Zymography Assays

6‐week‐old female C57BL/6 mice were injected with 4MOSC1 cells into the tongue. When tumors were palpable, the tumor and peri‐tumoral muscle were harvested and frozen in liquid nitrogen. Tissues were placed in cold NP‐40 lysis buffer (Invitrogen FNN0021) containing both phosphatase (Pierce 88667) and protease inhibitors (Millipore Sigma 4693124001), homogenized by passing through a syringe, centrifuged at 16 000 g for 10 min at 4 °C, and protein concentration measured on collected supernatants. 2x Tris Glycine SDS Sample Buffer (Novex LC2676) was added to the samples and equivalent amounts of total protein loaded onto 10% zymogram gelatin gels (Novex ZY00100BOX). Gels were developed using Novex zymogram renaturing (Novex LC2670) and developing (Novex LC2671) buffers (10X) and stained with SimplyBlue Safestain (Invitrogen LC6065). Purified MMP‐2 and MMP‐9 controls were loaded and run to identify standard locations of gelatinase activity.

### NanoString Analysis

RNA was isolated from cells or tumor tissues and gene expression profiling was analyzed using the NanoString PanCancer IO 360 Panel gene expression platform, n = 3 samples per group. For Data Preparation and Normalization, Bioinformatic analysis was performed using the Nanotube and NanoStringNCTools packages.^[^ ^54^ ^]^ Briefly, for each experiment, RCC files corresponding to each sample as well as sample metadata were uploaded into a single dataset using the processNanostringData function. Normalization was performed using the parameter ‘nSolver’ to obtain normalized expression values for each gene, source data provided. Next, correlation plots were generated based on normalized expression values for each sample. Principal Component Analysis (PCA) was performed using the nanostringPCA function based on the normalized expression values. Differential expression analysis was performed using limma via the Nanotube package.^[^ ^55^ ^]^ Differentially expressed genes were identified using the runLimmaAnalysis function, with base.group set to “Control”. For each contrast, significantly differentially expressed genes were selected based on thresholds of | log_2_FC | > 1 and log_10_(*p*‐value) > 2. Pathway analysis was conducted using the clusterProfiler package.^[^ ^56^, ^57^ ^]^ Based on differential expression results as described above, significant genes were selected. Gene names were converted to Entrez IDs, and pathway analysis was performed using the enrichGO function with the following parameters: OrgDb = ‘org.Mm.eg.db’, ont = ‘BP’, pAdjustMethod = ‘BH’, pvalueCutoff = 0.01, qvalueCutoff = 0.05. The Advanced Analysis module of the nSolver software was used to analyze genes associated with listed immune cells in tumors and given a Z‐score.

### Flow Cytometry Analysis

B16 tumors established in 6‐week‐old female C57BL/6 albino mice were treated with free CDX3379‐MMAE, CDX3379‐DXd, and IR as indicated in in Figure Legends, minced, and re‐suspended in FBS‐free DMEM media supplemented with components of MACs tumor dissociation kit (Miltenyi Biotec 130‐096‐730). Tissues were incubated for 15 min at 37 °C and mechanically digested using the gentle MACs Octo Dissociator. Tissue suspensions were washed with fresh media and passed through a 100‐µm strainer. Samples were washed with PBS and immediately processed for live/dead cell discrimination using LIVE/DEAD Fixable Blue Dead Cell Stain Kit (Invitrogen L2305). Cells were washed and stained for surface markers for 30 min at 4 °C. Intracellular staining was performed using the eBioscience FOXP3/Transcription Factor Staining Buffer Set (Invitrogen 00‐5525‐00) and stained with intracellular antibodies. Details for antibodies used in flow cytometry studies (supplier, clone, and catalog number) are listed in Figure S17 (Supporting Information). All antibodies were validated by the supplier. All flow cytometry data acquisition was done using BD LSRFortessa and analyzed using FlowJo software. Specific immune cells were identified by the following characteristics: CD8+ cells (CD45+Thy1.2+CD8+), memory CD8 T‐cells (CD44+ CD62L+), Tregs (Foxp3+ CTLA4+)

### Statistical Analysis

One‐way ANOVA with Tukey's or Dunnett's multiple comparisons test were performed for quantitative cell culture IC_50_ and end of study tumor volume responses. Survival curves were analyzed Log‐rank (Mantel‐Cox). All statistical analyses were performed using Prism software, version 10.0.3 (GraphPad).

## Conflict of Interest

S.R.A. and S.J.A.: Co‐inventor on University of California San Diego filed patent applications: “Tumor radiosensitization with monomethyl auristatin E (MMAE) and derivatives thereof” USPTO #10596259; S.J.A: “Tumor radiosensitization with antibody conjugates” US Patent Application No. 15/817,017 covering MMAE and ADC based radiosensitization, respectively. CDX3379 anti‐HER3 antibody for ADC synthesis was provided by Celldex Therapeutics.

## Supporting information



Supporting Information

## Data Availability

The data that support the findings of this study are available from the corresponding author upon reasonable request.
