# Supplementary material for: Interactions of Antibody Drug Conjugate Anti‐Tubulin and Topoisomerase I Inhibitor Payloads with Radiotherapy to Potentiate Immunotherapy
Source: Adv Sci (Weinh). 2025 Oct 24;13(35):e06552. doi: 10.1002/advs.202506552 (PMC13292169; doi:10.1002/advs.202506552)
Supplement: Supplementary file 1 — Supporting Information [file ADVS-13-e06552-s001.pdf]

Interactions of Antibody Drug Conjugate Anti-Tubulin and Topoisomerase I Inhibitor Payloads with Radiotherapy to Potentiate Immunotherapy

Jacqueline Lesperance, Bryan S. Yung, Michael M. Allevato, Marcus M Cheng, Maria F. Camargo, Robert Saddawi-Konefka, Kanika Dhawan, Ashwyn K. Sharma, Mahsa Mortaja, Sophie Bice, Daniel J. Scanderbeg, Diego Alvarado, Jyoti Mayadev, Ramez N. Eskander, Stephen R. Adams, Pippa F. Cosper, J. Silvio Gutkind, Sunil J. Advani*

Supplementary Information

Figure S1

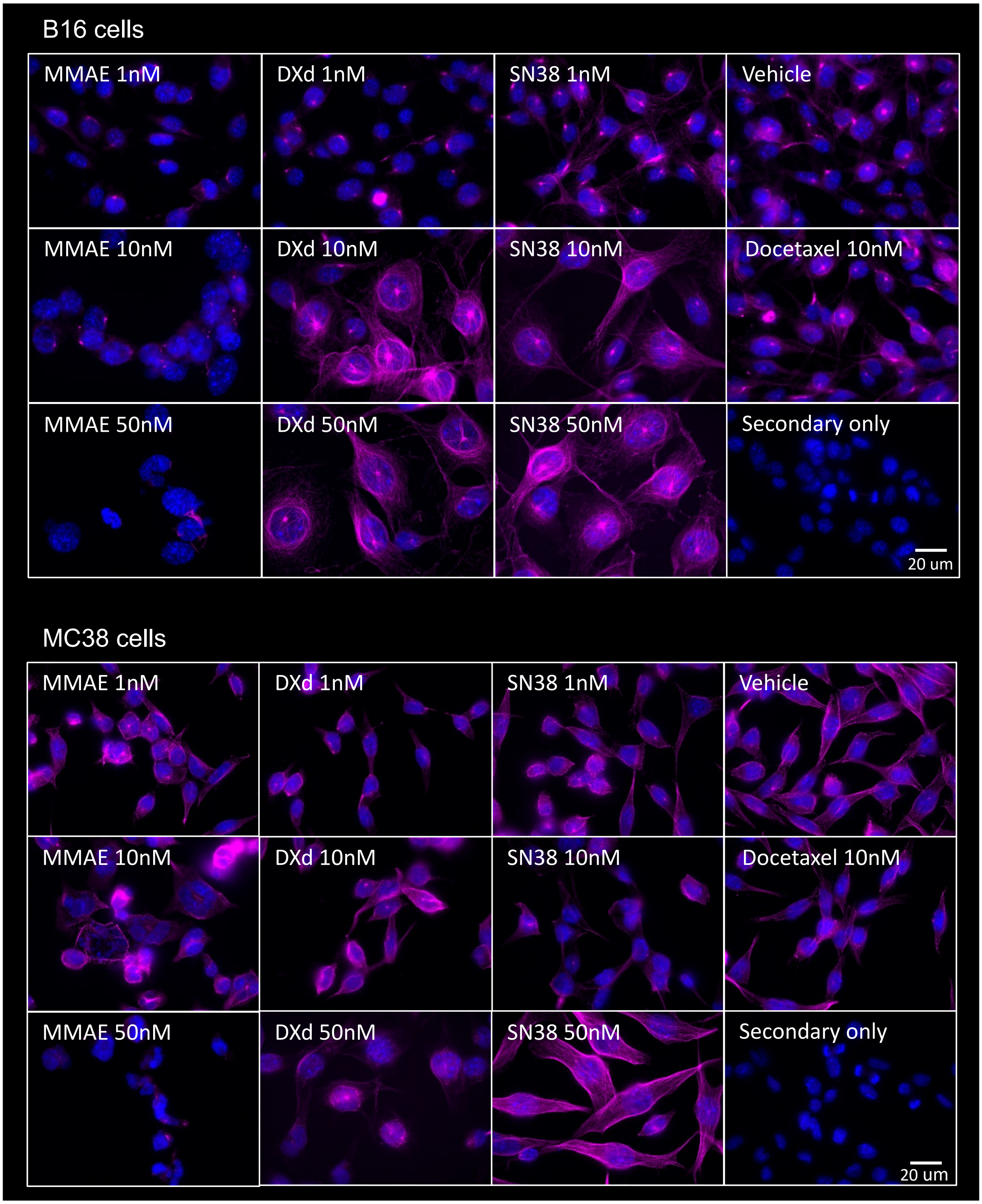

Figure S1: Tubulin staining of anti-tubulin and topoisomerase I inhibitor ADC drug payloads. B16 and MC38 cells treated with a dose range of MMAE, DXd and SN-38 for 24 hours. Docetaxel was used a positive control. Cells fixed and stained for α-tubulin staining (magenta). Nuclei counterstained with DAPI (blue).

Figure S2

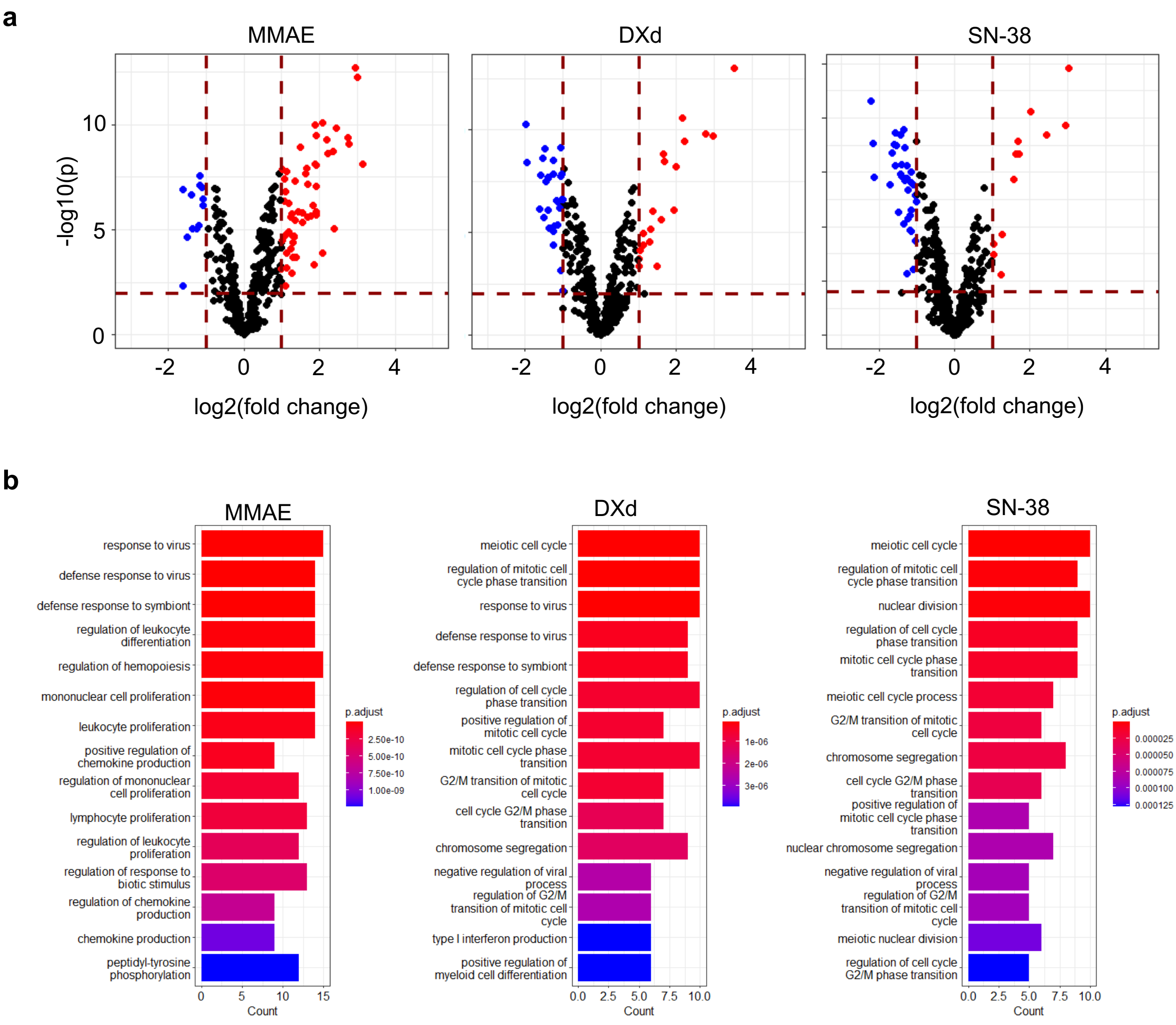

Figure S2: Gene expression alterations induced by anti-tubulin and topoisomerase I inhibitor ADC drug payloads. a) Volcano plots for gene expression of MMAE, DXd and SN-38 treated B16 cells vs control untreated samples, n=3. Dashed lines represent thresholds for significance in terms of p-value (horizontal lines) and log2 fold change (vertical lines). Red dots indicate significantly upregulated genes and blue dots indicate significantly downregulated genes in drug treated cells. Source data in Source Data file. **b)** Pathway analysis results for MMAE (left), DXd (center), and SN-38 (right) treated B16 cells vs control untreated samples, n=3. The top 15 biological processes from Gene Ontology that are enriched are displayed. Each bar depicts the enrichment scores (p-values) and gene count within the corresponding gene set as bar height.

Figure S3

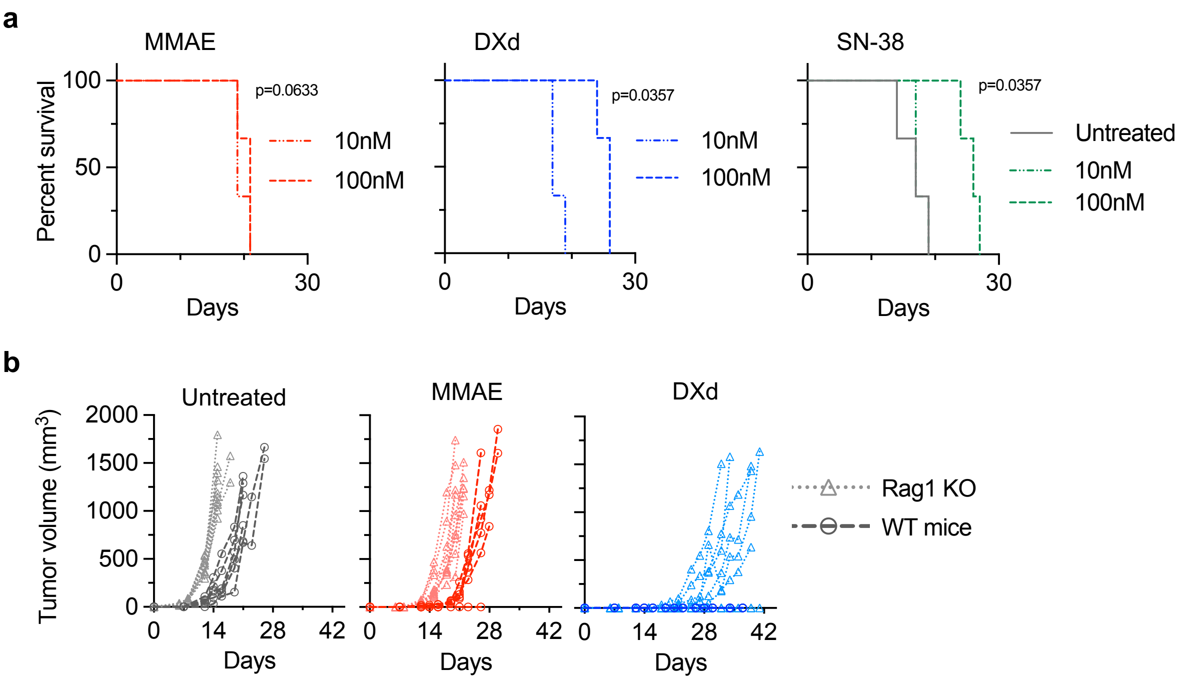

Figure S3: Cytotoxicity and adaptive immunity contribution on anti-tumor efficacy of anti-tubulin and topoisomerase I inhibitors. a) B16 cells exposed to 10 or 100 nM MMAE, DXd or SN-38 for 24 hours in cell culture and implanted into C57BL/6 mice. Mouse survival plotted and statistical significances calculated using Log-rank (Mantel-Cox) test, n=3. **b)** MC38 cells exposed to 100 nM MMAE or DXd for 24 hours in cell culture and implanted into WT or RAG1 knockout mice. Individual tumor volumes plotted over time, n=6 (WT), n=10, (RAG1 KO).

Figure S4

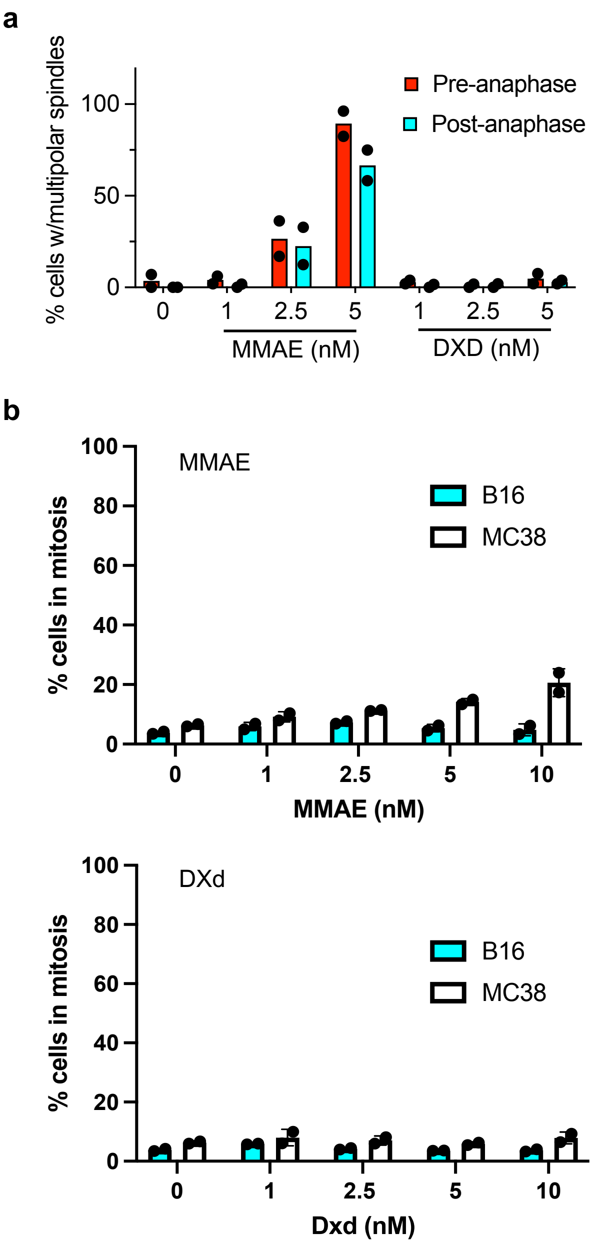

Figure S4: MMAE induces multipolar spindles independently of mitotic arrest, while Dxd does not. a) Quantification of multipolar spindles pre-anaphase (prometaphase or metaphase) and post-anaphase in B16 cells treated with dose range of MMAE or DXd for 24 hours, n=2. Data plotted as mean with scatter plot. At least 50 mitotic cells in each phase of mitosis counted per sample. Data plotted as scatter plot, n=2. **b)** Quantification of the percentage of cells in mitosis (mitotic index) in both MC38 and B16 cells treated with MMAE (upper panel) or Dxd (lower panel). At least 500 cells were counted per condition, n=2 biological replicates each.

Figure S5

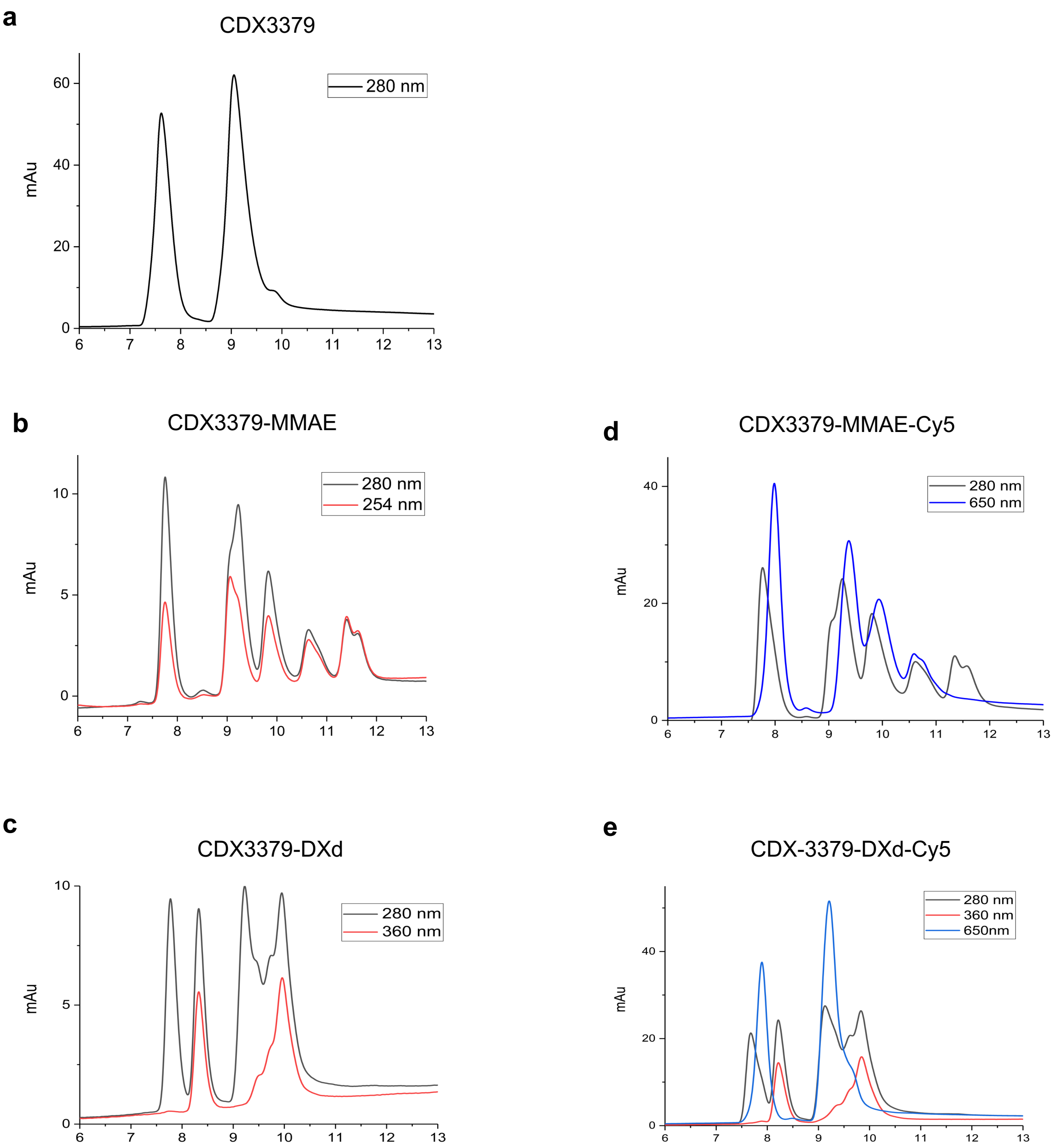

Figure S5: Reverse-phase HPLC chromatograms of CDX3379 before and after labeling with MMAE and DXd drug linkers. CDX3379 after reduction with excess DTT **(a)**, or with 4 equivalents of TCEP and subsequent reaction with 4 equivalents of **(b)** MC-VC-PABC-MMAE or **(c)** Deruxtecan. Subsequent reaction with 2 equivalents of Cy5-maleimide, followed by gel filtration and concentration gave conjugates with **(d)** MMAE and **(e)** DXd. Samples were treated with 50 mM DTT at 37°C for 15 min prior to analysis. HPLC separation on Agilent PLRP-S 1000A 8um column eluting with a 20-55% gradient of acetonitrile-water-0.05% TFA in 14 mins at 1ml/min at 90°C.

Figure S6

a

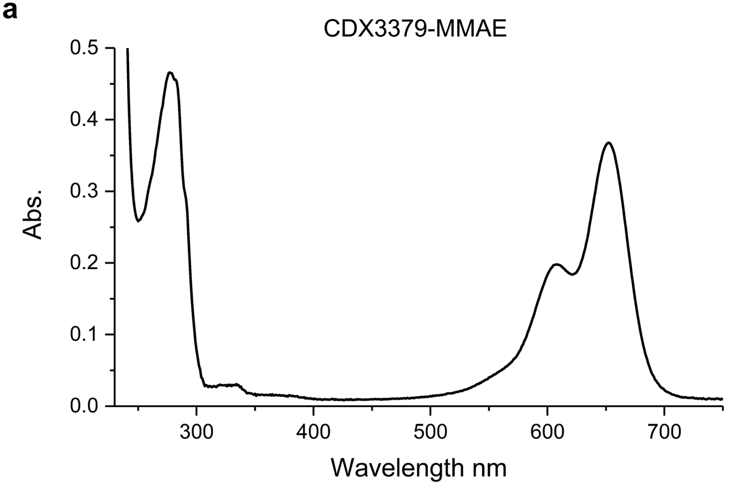

b

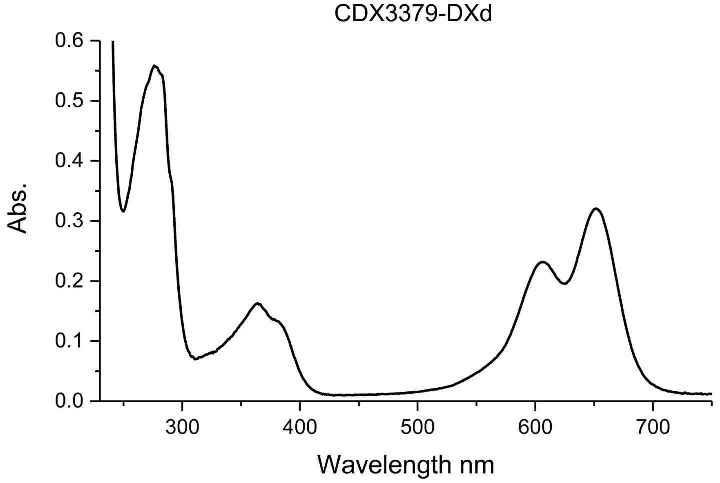

Figure S6: UV-visible absorbance spectra of Cy5 labeled ADCs. Anti-HER3 CDX3379 antibody ADC in PBS, with **(a)** MC-VC-PABC-MMAE and Cy5 or **(b)** Deruxtecan and Cy5. Absorbance peaks at 650, 360 and 280 nm are used to determine labeling stoichiometries of Cy5, Deruxtecan, and antibody concentration.

Figure S7

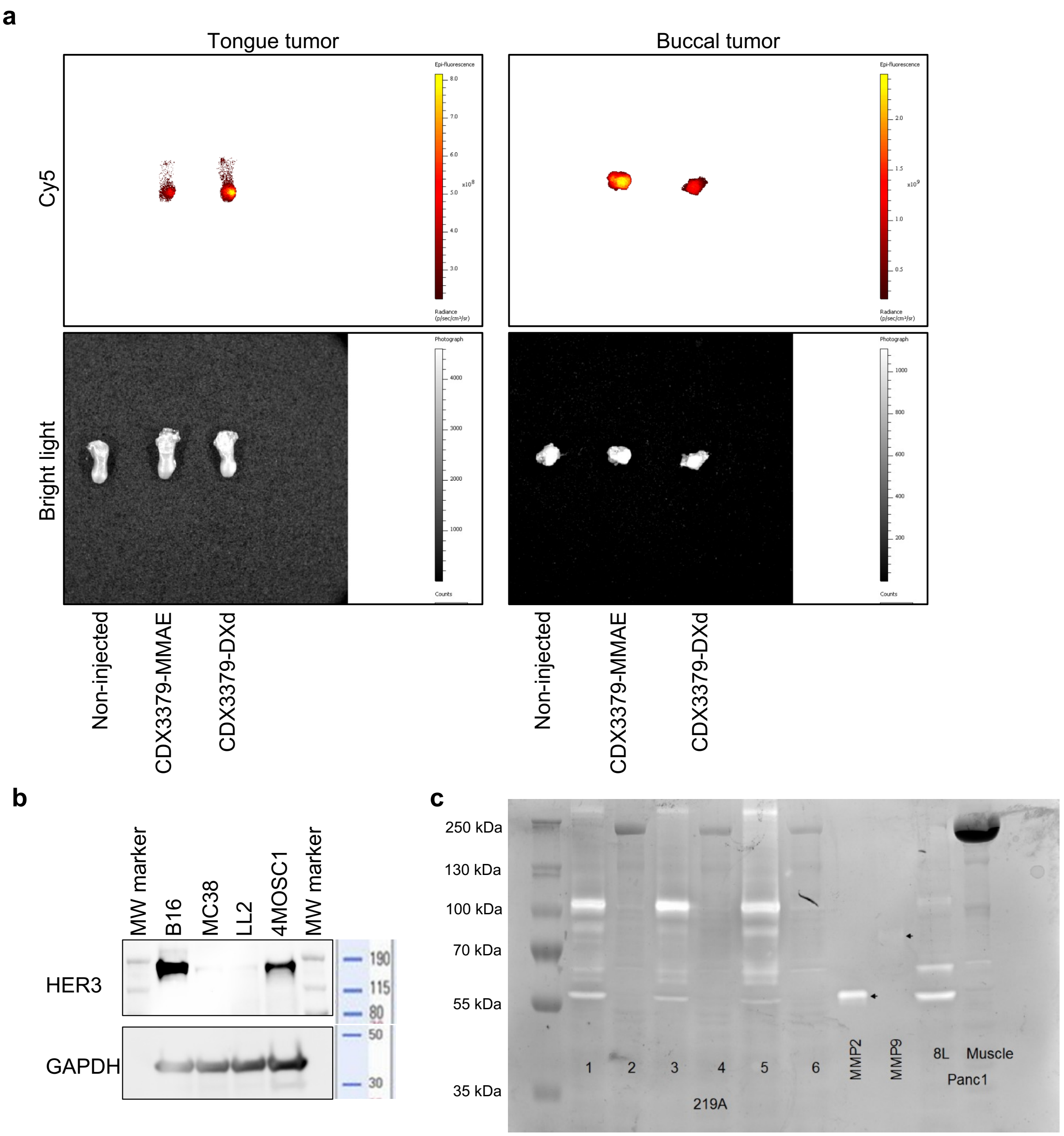

Figure S7: Targeting specificity of synthesized ADCs and ACPPs. a) Tumor-targeted ADC localization. 2.5 nmol Cy5 labeled CDX3369-MMAE or CDX3379-DXd HER3 targeted ADC i.v. injected into mice with orthotopic tongue or cheek implanted 4MOSC1 tumors. Whole tongue or buccal tissue resected and imaged by bright light and Cy5 48 hours later, pseudocolor scale bars on right of each image. Uncropped image of data shown in Figure 4d. **b)** HER3 expression in murine cancer cell lines. Immunoblot for total HER3 and GAPDH. MW marker ladder in left lane. Uncropped blot of data shown in Figure 4c. **c)** Gelatinase matrix metalloproteinase activity in tumor tissue. Gelatin zymography of murine 4MOSC1 tumors and adjacent normal murine tongue tissue from orthotopically grown tumors. Uncropped blot of data shown in Figure 4e (lanes marked 1-4). Molecular weight marker (MW) in far-left lanes. Control MMP-2 and MMP-9 activity in marked lanes and activity depicted by black arrows within blot.

Figure S8

a

ACPP-MMAE

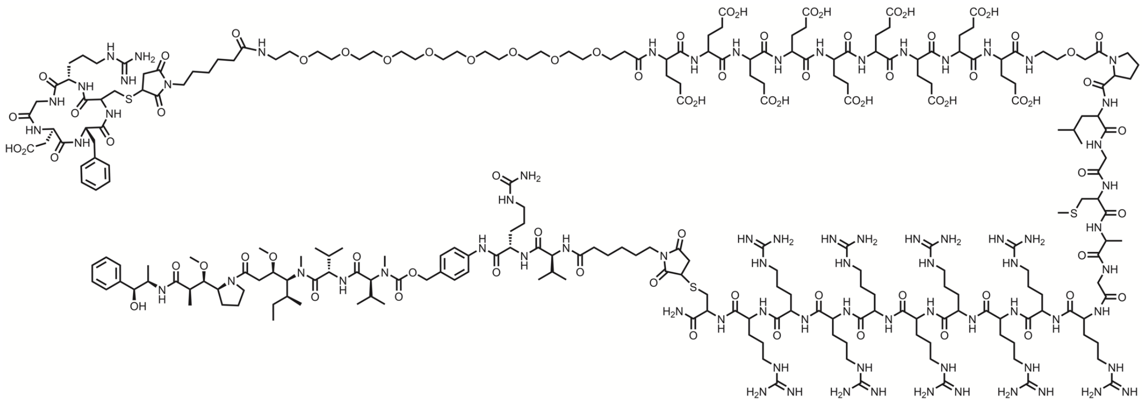

b

ACPP-DXd

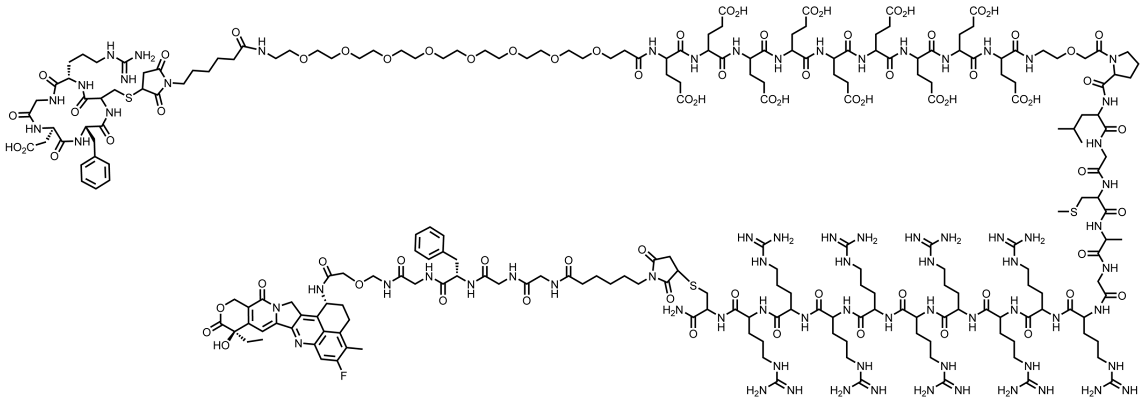

Figure S8: Chemical structures of ACPP-drug conjugates. ACPP with cRGD pretargeting attached to **(a)** MMAE or **(b)** DXd using clinical ADC drug-linker chemistry combinations.

Figure S9

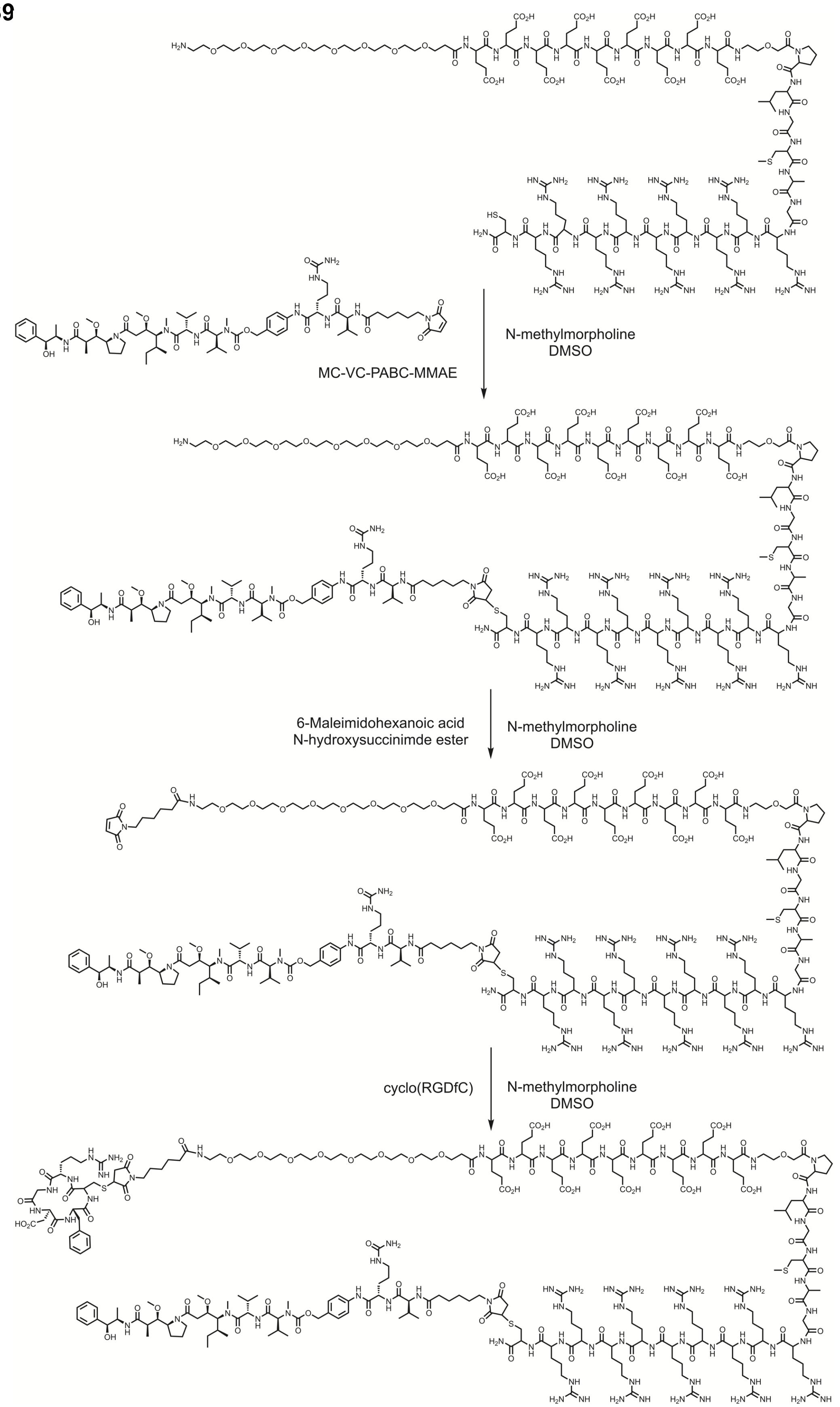

Figure S9: Synthetic scheme for ACPP-MMAE. A one-pot synthesis from ACPP in DMSO and N-methyl morpholine by successive additions of drug linker MC-VC-PABC-MMAE to the C-terminal cysteine, 6-maleimidohexanoic acid N-hydroxysuccinimide ester to the N-terminal amino group, and cyclo(RGD)fC to the maleimide group.

Figure S10

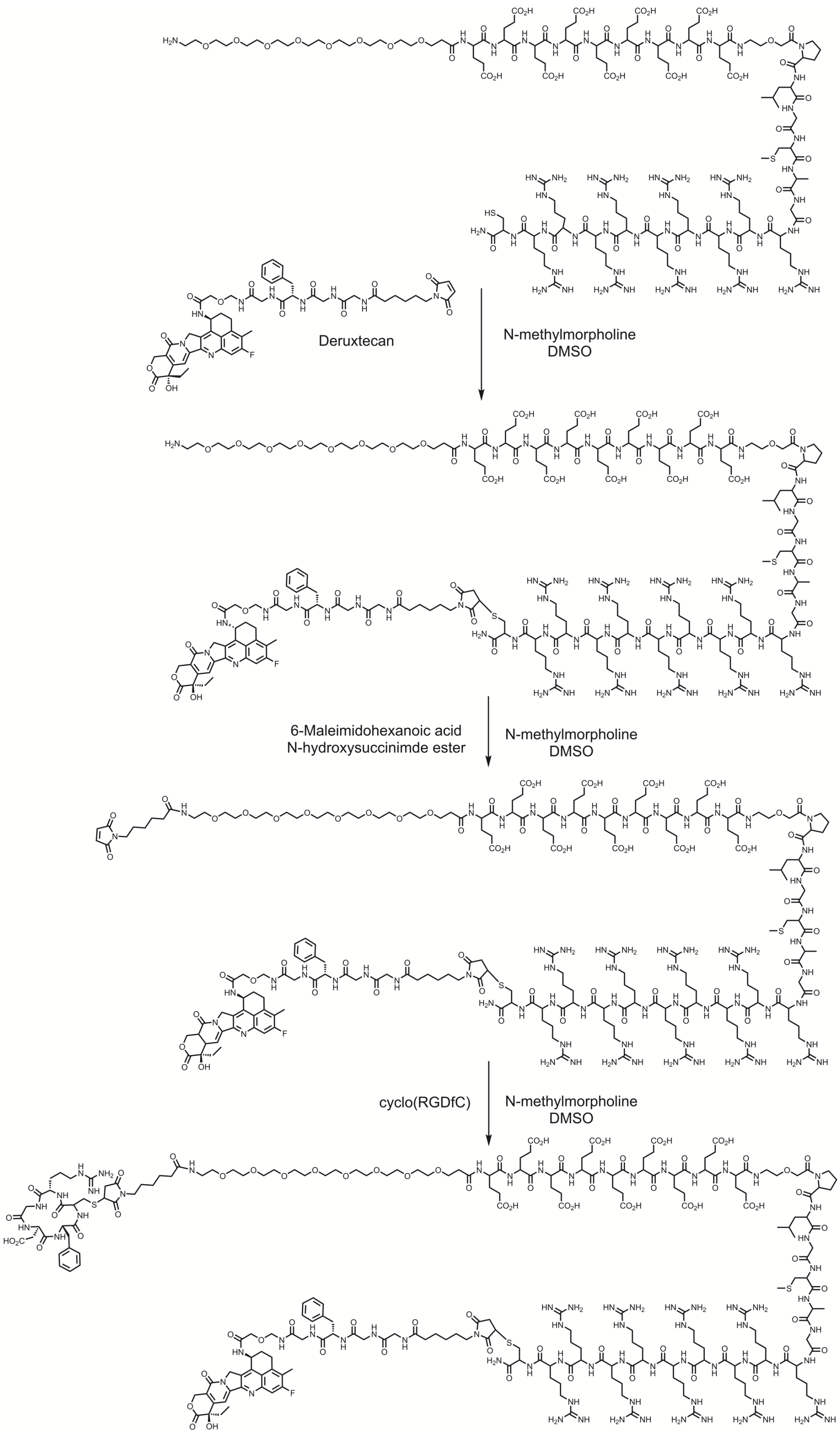

Figure S10: Synthetic scheme for ACPP-DXd. A one-pot synthesis from ACPP in DMSO and N-methyl morpholine by successive additions of drug linker Deruxtecan to the C-terminal cysteine, 6-maleimidohexanoic acid N-hydroxysuccinimide ester to the N-terminal amino group, and cyclo(RGD)fC to the maleimide group.

Figure S11

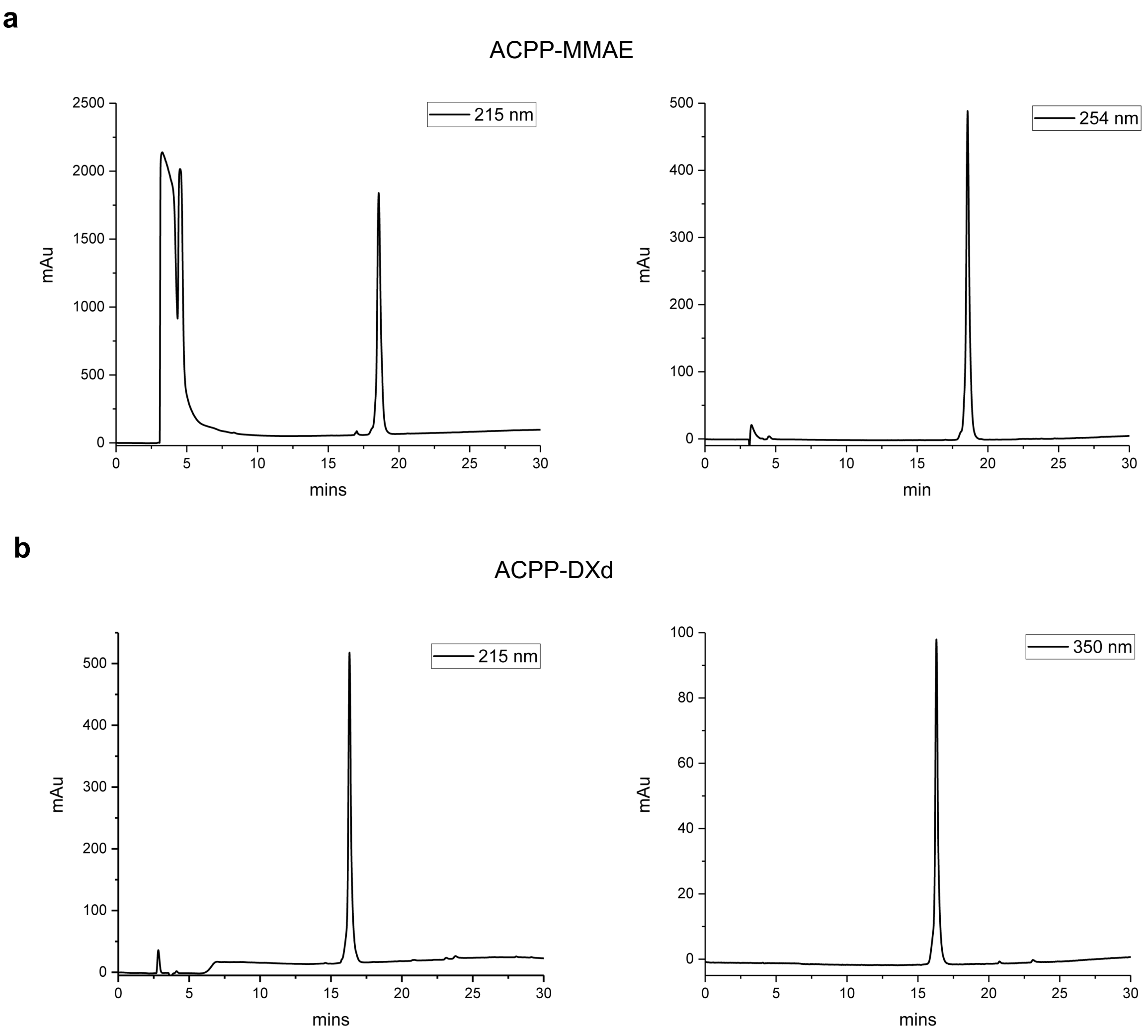

Figure S11: Reverse-phase HPLC chromatograms of ACPP drug conjugates. a) ACPP-MMAE, monitored at 215 nm (left trace) and 254 nm (right trace). The sample was diluted in DMSO that gave the peaks eluting at 4-5 min. **b)** ACPP-DXd, monitored at 215 nm (left trace) and 360 nm (right trace).

Figure S12

a

ACPP-MMAE, $C_{249}H_{409}N_{75}O_{79}S_3$

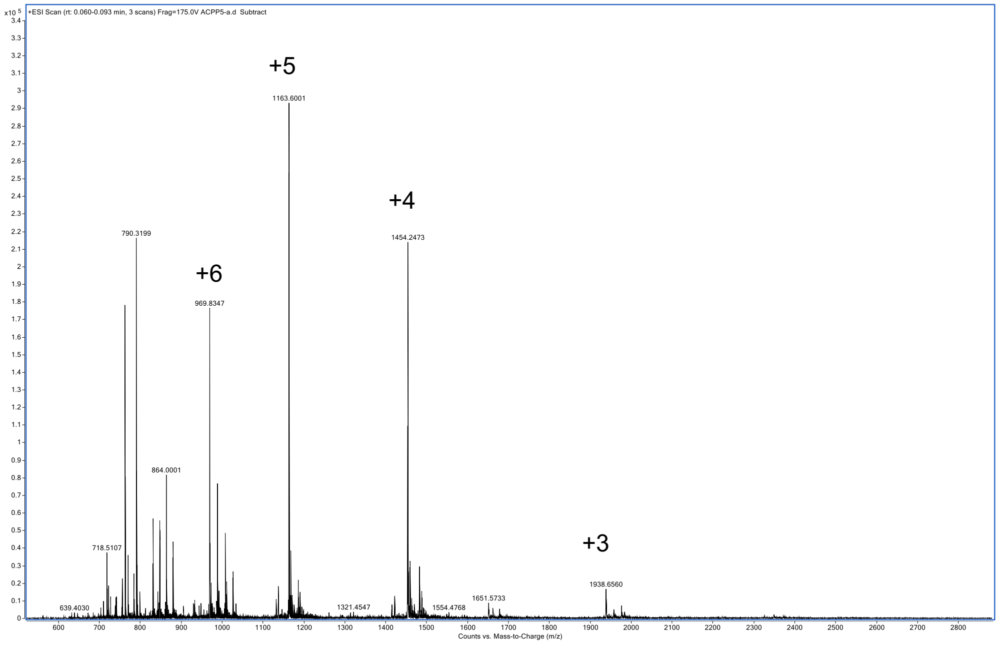

b

Calculated $m_{average}$ 5813.5711 Da
Found: 5812.9

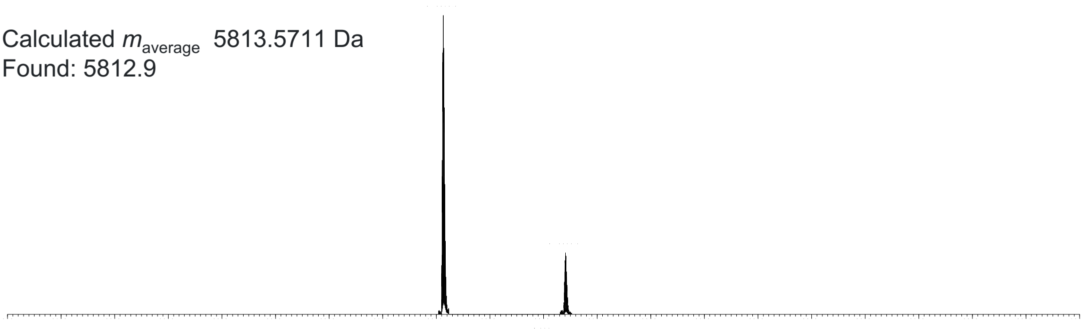

c

Calculated $m_{monoisotopic}$ 5809.9455 Da
Found: 5809.912

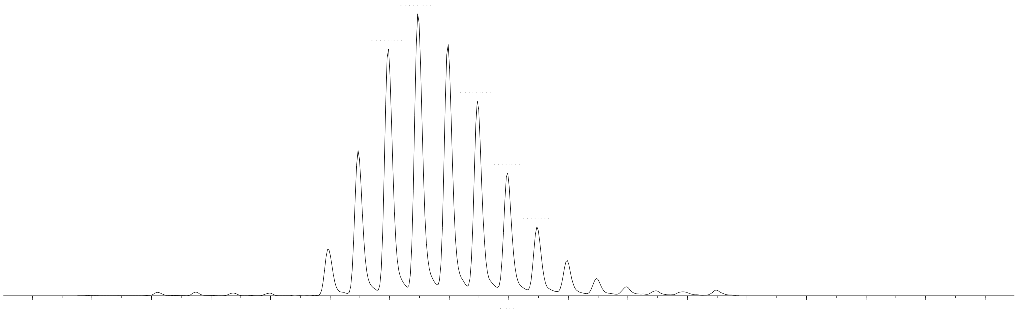

Figure S12; High resolution mass spectroscopy of ACPP-MMAE. a) ESI-TOFM (Agilent LC-Agilent 6230 ESI-TOFMS) mass/charge spectrum in positive mode with multiple charged peaks marked. **b)** Deconvolved mass spectrum, and **c)** expanded region of deconvolved mass spectrum with found and calculated (Prot-Pi) average and monoisotopic masses.

Figure S13

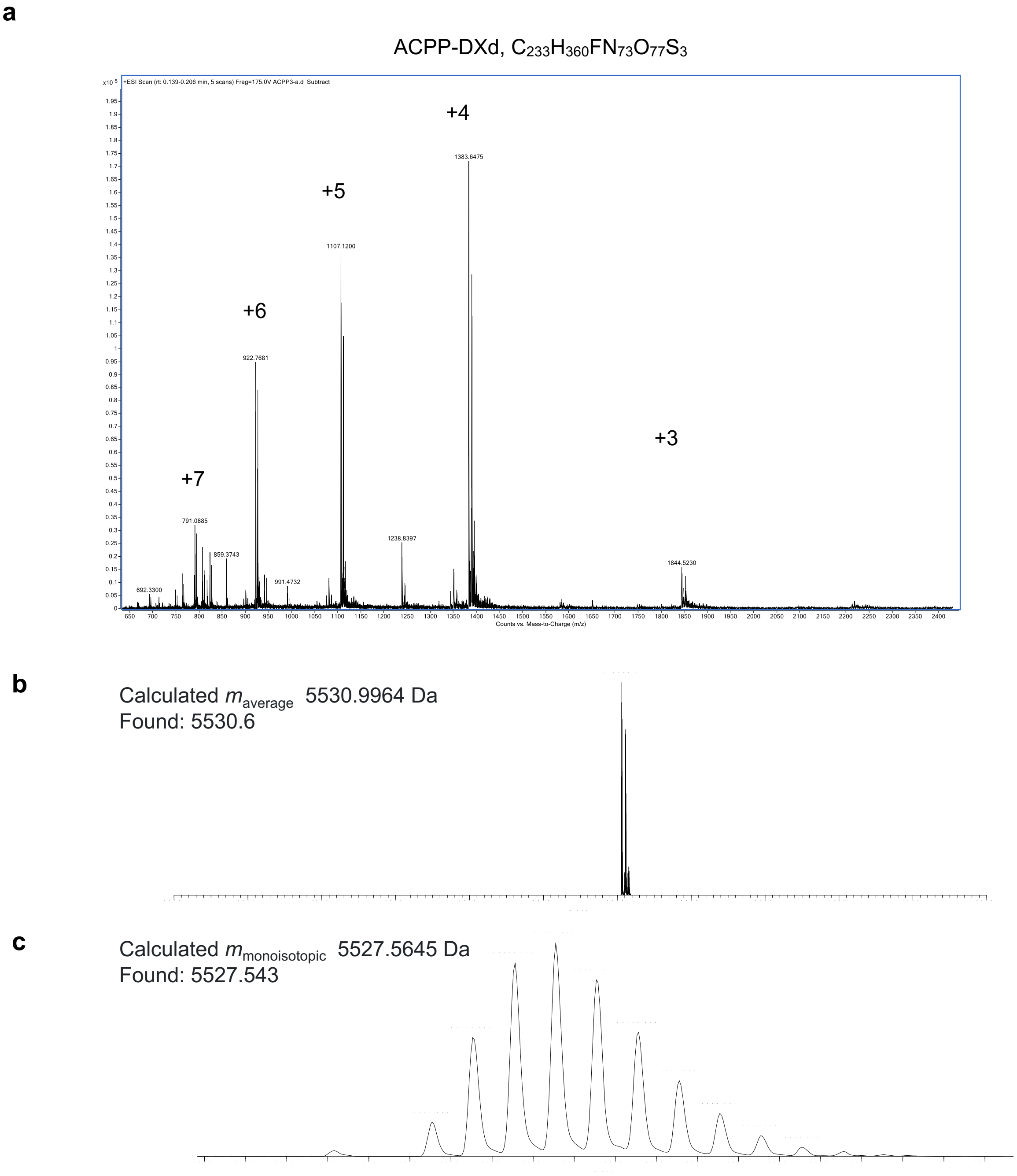

Figure S13: High resolution mass spectroscopy of ACPP-DXd. a) ESI-TOFM (Agilent LC-Agilent 6230 ESI-TOFMS) mass/charge spectrum in positive mode with multiple charged peaks marked. **b)** Deconvolved mass spectrum, and **c)** expanded region of deconvolved mass spectrum with found and calculated (Prot-Pi) average and monoisotopic masses.

Figure S14

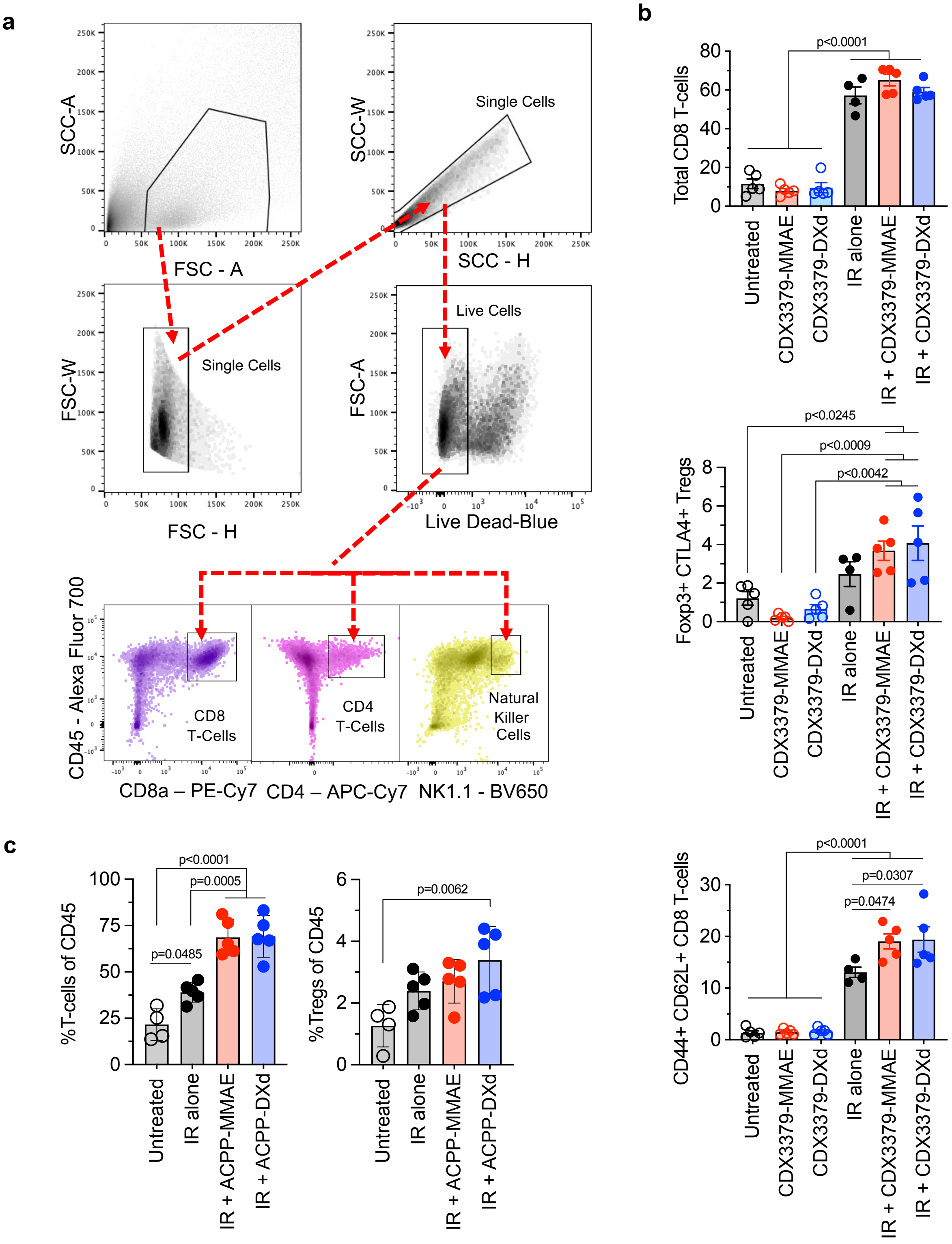

Figure S14: Irradiated tumor immune infiltration alterations induced by anti-tubulin and topoisomerase I inhibitors. a) Representative flow cytometry gating strategy to gate tumor-infiltrating immune cells. **b)** Mice with B16 tumors treated with CDX3379-MMAE or CD3379-DXd and IR as in Figure 5b. Tumors harvested on day 18 and immune cell type abundance measured by flow cytometry, n=5 except for IR alone (n=4). Data normalized as % of CD45 cells and plotted as scatter plot with mean ± SEM. Statistical significances calculated using one-way ANOVA with Tukey's multiple comparisons test. **c)** Mice with B16 tumors treated with ACPP-MMAE or ACPP-DXd and IR as in Figure 5c. Tumors harvested on day 18 and immune cell type abundance measured by flow cytometry, n=5 except for untreated (n=4). Data normalized as % of CD45 cells and plotted as scatter plot with mean ± SEM. Statistical significances calculated using one-way ANOVA with Tukey's multiple comparisons test.

Figure S15

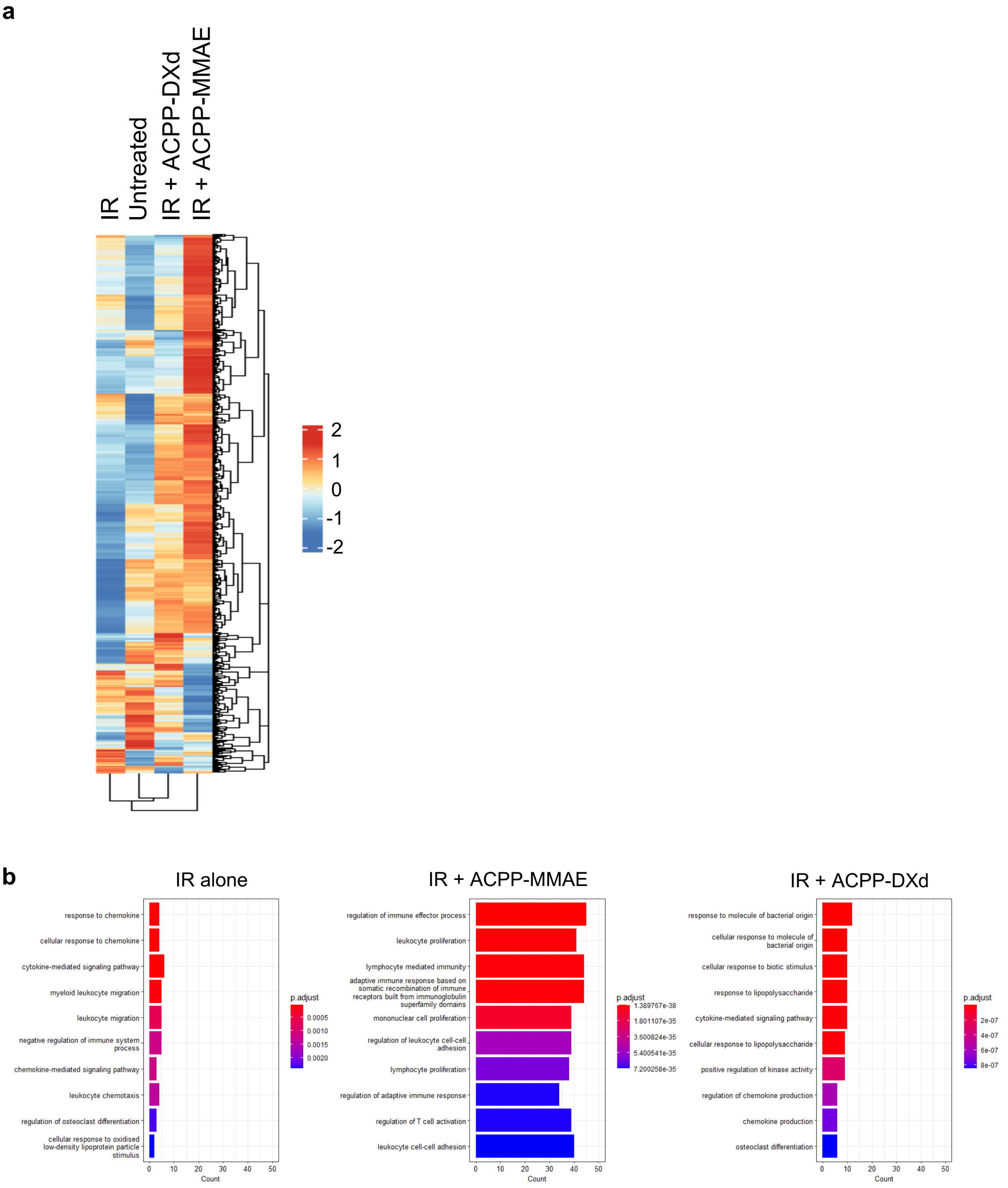

Figure S15: Irradiated tumor immune microenvironment gene expression alterations induced by anti-tubulin and topoisomerase I inhibitors. a) Heatmap of gene expression for all samples. Normalized gene expression values (ranging from -2 to 2) between samples for all endogenous (non-housekeeping) genes within the Nanostring nCounter assay. Normalized expression values were then averaged by condition for all samples, n=3. Source data in Source Data file. **b)** Pathway analysis results for IR alone (left), IR + ACPP-MMAE (center), and IR + ACPP-DXd (right) treated B16 tumors vs control untreated samples, n=3. The top 10 biological processes from Gene Ontology that are enriched are displayed. Each bar depicts the enrichment scores (p-values) and gene count within the corresponding gene set as bar height.

Figure S16

a

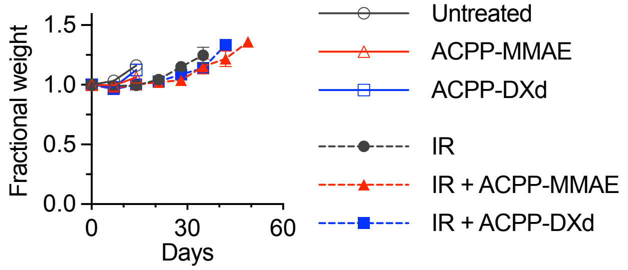

b

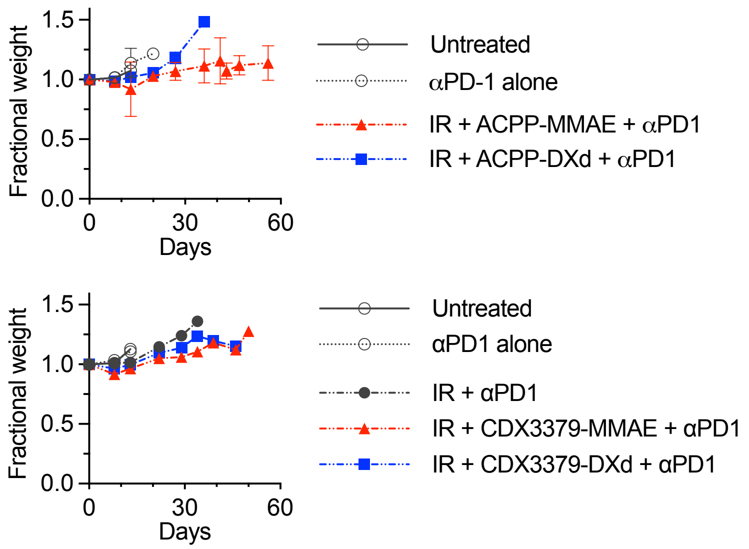

Figure S16: Safety of tumor targeted MMAE with IR. a) Mouse body weights of mice from experiments in Figure 5f. Mouse body weight were normalized to each mouse's weight on day 0, i.e. tumor implantation date. Data plotted as mean fractional body weight ± SEM. **b)** Mouse body weights of mice from experiments in Figures 5g, 5h were normalized to each mouse's weight on day of tumor implantation. Data is plotted as mean fractional body weight ± SEM.

Figure S17

Marker Table					
Marker	Fluorescent Tag	Clone	Supplier	Size	Catalog #
**XCR1**	Spark UV 387	ZET	BioLegend	100 µg	148236
**Viability**	Ghost Dye UV 450		Cytek Biosciences	500 Tests	13-0868-T500
**CD8**	BUV496	53-6.7	BD Biosciences	50 µg	750024
**KLRG1**	BUV563	2F1	BD Biosciences	50 µg	741343
**CD19**	BUV661	1D3	BD Biosciences	50 µg	612971
**CD103**	BUV737	2E7	BD Biosciences	50 µg	749393
**Arginase-1 (Arg-1)**	BUV805	A1exF5	Thermo Fisher Scientific	100 µg	368-3697-82
**CD279 (PD-1)**	BV421	29F.1A12	BioLegend	100 µg	135218
**Ly-6C**	eFluor 450	HK1.4	Thermo Fisher Scientific	100 µg	48-5932-82
**CD80**	BV480	16-10A1	BD Biosciences	50 µg	746775
**CD45**	BV510	30.F11	BioLegend	50 µg	103138
**CD44**	BV570	IM7	BioLegend	125 µL	103037
**CD11c**	BV605	N418	BioLegend	50 µg	117334
**CD90.2 (THY1.2)**	BV650	53-2.1	BD Biosciences	50 µg	740442
**CD366 (TIM3)**	BV711	RMT3-23	BioLegend	50 µg	119727
**F4/80**	BV750	T45-2342	BD Biosciences	50 µg	747295
**CD223 (LAG-3)**	BV785	C9B7W	BioLegend	50 µg	125219
**TCF1 (TCF7)**	Alexa Fluor 488	C63D9	Cell Signaling	100 µL	#6444
**MHC Class II (I-A+I-E)**	Spark Blue 550	M5/114.15.2	BioLegend	100 µg	107662
**CD62L (L-Selectin)**	NovaFluor Blue 610	MEL-14	Thermo Fisher Scientific	25 µg	M006T02B04
**CD11b**	PerCP-Cy5.5	M1/70	BioLegend	100 µg	101228
**CD206 (MMR)**	PerCP-eFluor 710	MR6F3	Thermo Fisher Scientific	100 µg	46-2061-82
**FOXP3**	PE	MF-14	BioLegend	100 µg	126404
**Granzyme B**	PE-Dazzle594	QA16A02	BioLegend	100 tests	372216
**CD152 (CTLA-4)**	PE-Fire 640	UC10-4B9	BioLegend	100 µg	106334
**CD4**	PE-Fire 700	GK1.5	BioLegend	100 µg	100484
**CD161 (NK1.1)**	PE-Fire 810	PK136	BioLegend	25 µg	108767
**CD274 (PD-L1)**	APC	10F.9G2	BioLegend	100 µg	124312
**TOX**	Alexa Fluor 647	NAN448B	BD Biosciences	100 µg	568356
**Ki-67**	Alexa Fluor 700	16A8	BioLegend	100 µg	652420
**iNOS**	APC-eFluor780	CXNFT	Thermo Fisher Scientific	100 µg	47-5920-82
**Ly-6G**	APC-Fire 810	1A8	BioLegend	100 µg	127670

Figure S17: Antibodies use in flow cytometry studies.
